# Noncovalent Interactions in Halogenated Pyridinium Salts of the Weakly Coordinating Anion [Al(OTeF_5_)_4_]^−^

**DOI:** 10.1002/chem.202202749

**Published:** 2022-12-08

**Authors:** Sofiya Kotsyuda, Ahmet N. Toraman, Patrick Voßnacker, Mathias A. Ellwanger, Simon Steinhauer, Carsten Müller, Sebastian Riedel

**Affiliations:** ^1^ Fachbereich Biologie, Chemie, Pharmazie Institut für Chemie und Biochemie - Anorganische Chemie Fabeckstraße 34/36 14195 Berlin Germany

**Keywords:** anion-π interactions, fluorine-specific interactions, noncovalent interactions, weakly coordinating anion

## Abstract

The synthesis and the first structural characterization of the halogenated pyridinium salts [C_5_F_5_NH]^+^, [C_5_F_4_ClNH]^+^, [(C_5_F_5_N)_2_H]^+^, [(C_5_Cl_5_N)_2_H]^+^ of the weakly coordinating anion (WCA) [Al(OTeF_5_)_4_]^−^, showing noncovalent interactions in the solid state, are presented. The salts were characterized by the multinuclear NMR and IR spectroscopy as well as X‐ray diffraction. Hirshfeld surface analysis and solid state structures reveal various intermolecular anion‐π and σ‐hole interactions between the corresponding halogenated pyridinium cations and the anion [Al(OTeF_5_)_4_]^−^.

## Graphical abstract

The synthesis and structural characterization of the perhalogenated pyridinium cations [C_5_F_5_NH]^+^ and [C_5_F_4_ClNH]^+^ and the dimeric cations [(C_5_F_5_N)_2_H]^+^ and [(C_5_Cl_5_N)_2_H]^+^ as [Al(OTeF_5_)_4_]^−^ salts are presented. These cations show noncovalent interactions with the weakly coordinating anion [Al(OTeF_5_)_4_]^−^ such as strong fluorine specific interactions as well as σ‐hole and anion‐π interactions.

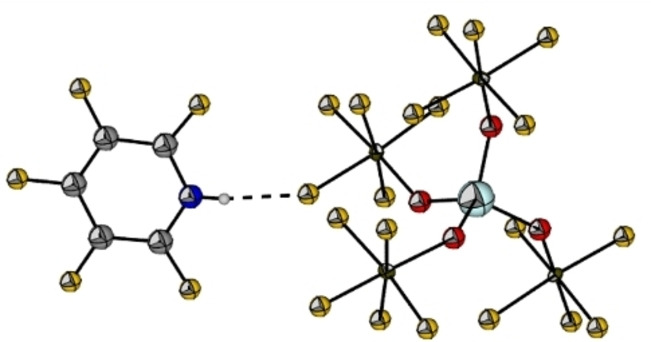

## Introduction

In contrast to pyridine, the perfluorinated pyridine C_5_F_5_N, exhibits an highly reduced basicity. While non fluorinated pyridine can be protonated by HCl or HBr, pentafluoropyridine can only be protonated by Brønsted superacids such as HF/AsF_5_ and HF/SbF_5_.^[1]^ The strong Brønsted superacid [*o*‐C_6_H_4_F_2_‐H][Al(OTeF_5_)_4_] is known for the protonation of weak bases like benzene and white phosphorus, resulting in [C_6_H_7_]^+^ and [P_4_H]^+^, respectively.^[2]^ This superacid can be potentially used for protonation of halogenated pyridines. Moreover, the perhalogenated pyridinium cations are strong acids themself and should give rise to a wide variety of binding types. Namely the N−H bond, which is a strong hydrogen bond donor and the halogen‐carbon bond that serve as halogen bond donor. Additionally, perhalogenated pyridines exhibit positive quadrupolar moments like in C_6_F_6_, which can result in anion‐π interactions.^[3]^ In contrary to the well‐known cation‐π interactions, anion‐π interactions are extremely rare and far less investigated.^[4]^ Anion‐π interactions occur between electron‐deficient arenes with a positive quadrupole moment and negatively charged or electron‐rich sites.^[3]^ They are directional and located above the aromatic ring periphery as a short contact and are predominantly investigated in the solid state.^[5]^ Previous studies showed anion‐π interactions between non‐fluorinated nitrogen‐containing hetrerocycles and small weakly coordinating anions (WCAs) like [BF_4_]^−^ and [PF_6_]^−[6]^ or between perfluoroarenes, like C_5_F_5_N, C_6_F_6_ and C_10_F_8_ with different halide anions.^[7]^ In this work, we investigated the protonation of C_5_F_5_N, C_5_F_4_ClN, and C_5_Cl_5_N by [*o*‐C_6_H_4_F_2_‐H][Al(OTeF_5_)_4_] (**1** **a**) and studied hydrogen bonding, halogen bonding and anion‐π interactions between pentafluororopyridinium [C_5_F_5_NH]^+^ (**2**), 4‐chloro‐2,3,5,6‐tetrafluoropyridinium [C_5_F_4_ClNH]^+^ (**3**), pentachlororopyridinium [C_5_Cl_5_NH]^+^ (**4**) and the fluorinated WCA [Al(OTeF_5_)_4_]^−^ (**a**).

## Results and Discussion

**Figure 1:**
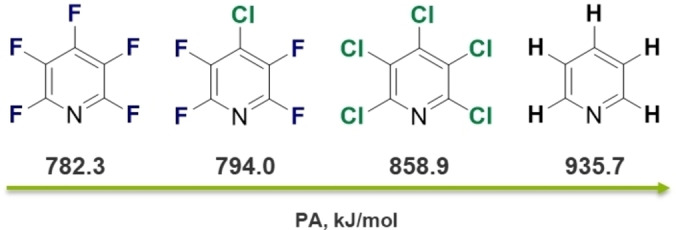
Proton affinities of C_5_F_5_N, C_5_F_4_ClN, C_5_Cl_5_N and C_5_H_5_N in kJ/mol, calculated on the B3LYP‐D3/def2‐TZVPP level of theory.

In order to study the basicity of some easily available perhalogenated pyridines, we calculated their proton affinities. As expected, the basicity trend follows the row C_5_F_5_N<C_5_F_4_ClN<C_5_Cl_5_N (Figure 1). Their proton affinities (PAs) are higher than for ortho‐difluorobenzene (743.0 kJ/mol) and therefore all of the pyridine derivatives can be in principle protonated by the superacid **1** **a**.

**Figure 2:**
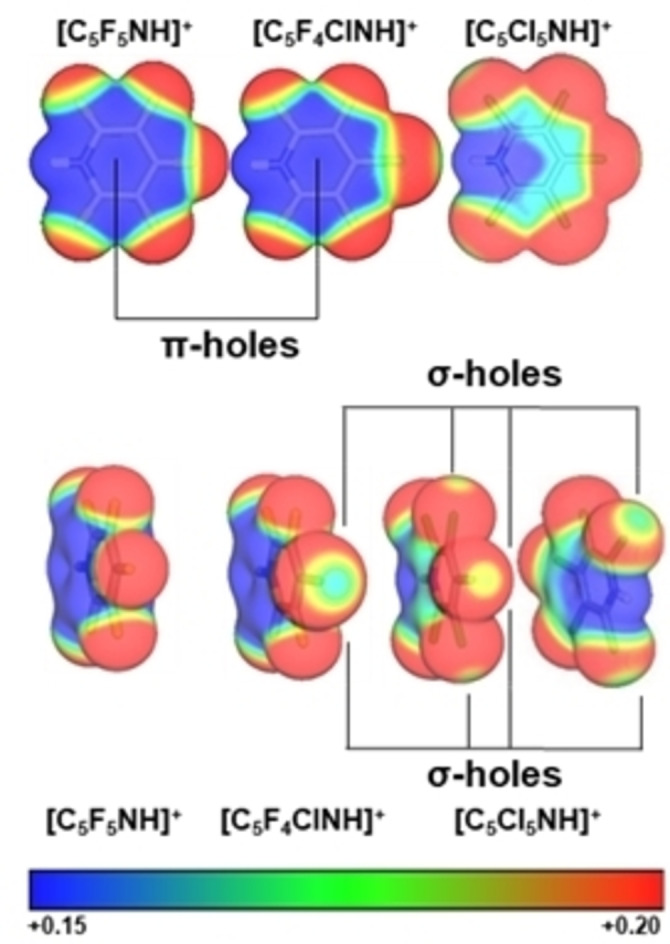
Computed electrostatic potentials of **2**, **3**, and **4** in the range of 0.15 e bohr^−3^ (red) to 0.20 e bohr^−3^ (blue) mapped onto their electron densities (isosurface value 0.0035 e bohr^−3^; B3LYP‐D3/def2‐TZVPP level of theory.

The computed plots of the electrostatic potentials (ESP) of the pyridinium cations illustrate that in all cases, their strongest interaction with an anion will be observed via the N−H moiety (Figure 2). Beside the hydrogen bond site, there is the region of depleted electron density above and below the aromatic pyridinium ring. This site is also known from the literature as π‐hole and can lead to anion‐π interactions.^[8]^ While the cations **2** and **3** show a strong depletion of the π‐electron density above the π‐bonds in a plane perpendicular to the pyridinium ring, for **4** only a weak depletion is observed. The chlorinated pyridinium cations **3** and **4** show additionally σ‐holes – an area of enhanced positive electrostatic potential, located on the carbon‐chlorine axis.^[9]^ For the cation **4** differently sized σ‐holes on the chlorine atoms are observed, see Figure 2. The biggest σ‐holes are found for the 2,6‐position. In salts with cation **3**, it might be possible to study all above discussed interactions simultaneously.

**Scheme 1:**
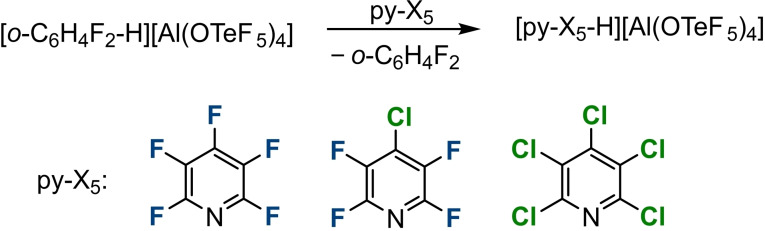
Formation of the halogenated pyridinium salts by the proton transfer reaction from the Brønsted superacid **1** **a**.

In order to obtain the corresponding pyridinium salts, we treated C_5_F_5_N, C_5_F_4_ClN or C_5_Cl_5_N with the Brønsted superacid **1** **a** in *ortho*‐difluorobenzene (*o*DFB) (Scheme 1).

**Figure 3:**
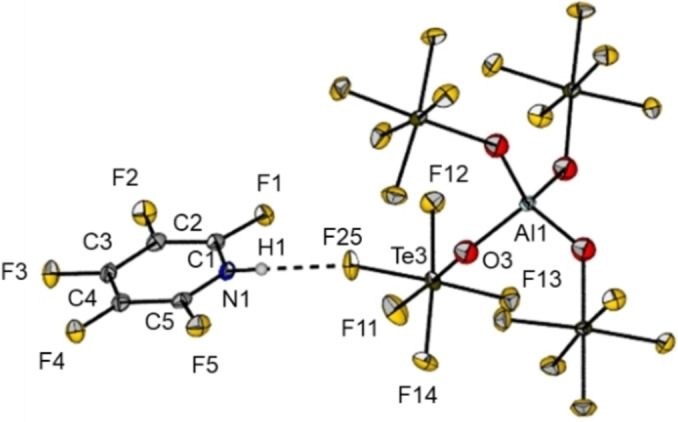
Molecular structure of **2** **a** in the solid state with thermal ellipsoids shown at 50 % probability level. The closest contact (F25‐N1; dashed line) shows a distance of 270.82(25) pm.

The salt **2** **a** crystallizes in the triclinic space group *P* $\bar{1}$ (Figure 3) by slowly cooling down the reaction mixture to −24 °C. The short N⋅⋅⋅F distance of 270.82(25) pm, is in agreement with a strong hydrogen bond between the N−H moiety and a fluorine atom of the WCA.^[10]^

**Figure 4:**
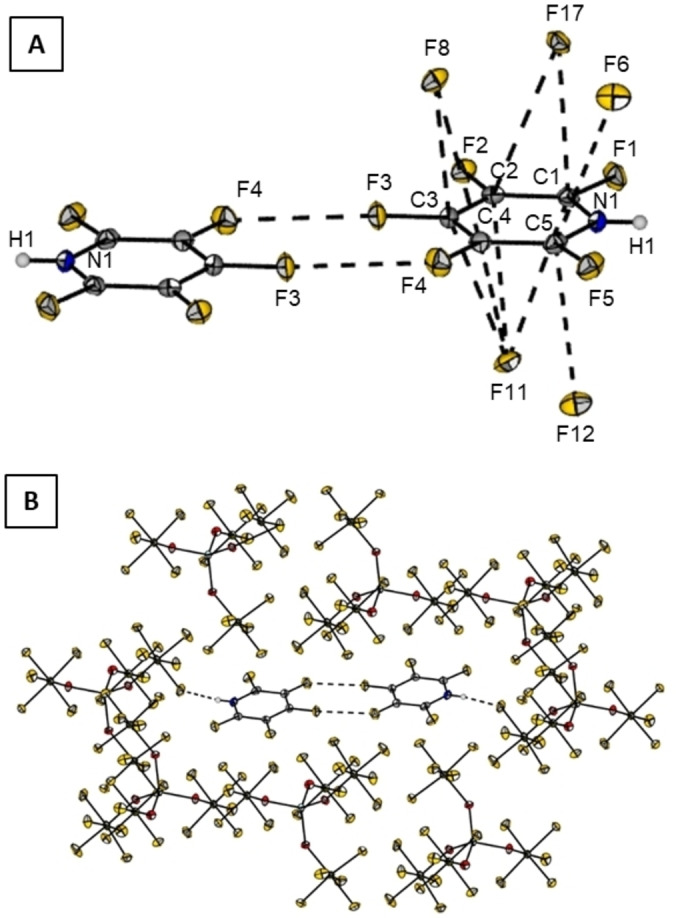
Crystal packing of **2** **a** (B) with a closer view on short contacts between two cationic moieties **2** (A). Dashed lines stand for distances [pm] of: F3*‐*F4 276.8(21), F8*‐*C3 290.2(20), F8*‐*C4 304.99(19), F11*‐*C4 317.61(21), F11*‐*C3 301.47(19), F11*‐*C2 302.90(19), F11‐C1 317.61(22), F12*‐*C5 294.06(19), F17‐C2 303.58(22), F17‐C1 282.32(19), and F12*‐*C5 294.06(19).

Figure 4 shows the co‐planar arrangement of two neighboring moieties of **2** with a C3‐F3‐F4‐C4 dihedral angle of 180(2)°. These moieties are symmetry related by an inversion center. As short fluorine/fluorine (F4⋅⋅⋅F3) contacts of 276.8(21) pm are observed between the two cations we further inspected the fluorine‐specific interaction in an isolated [C_5_F_5_NH]^+^‐dimer by means of atoms in molecules (AIM) analysis. At the bond critical points (CP) between F3 and F4 the electron density is very low (ρ_CP_=0.045 e A^−3^) and the Laplacian is small and positive (▿^2^ρ_CP_=0.035 e A^−5^). Together with a value of the electron localization function (ELF) close to zero (0.01) and a ratio of the absolute potential (|V|) and the kinetic energy density (G) below 1 (0.74), this indicates a non‐shared interaction such as a van‐der‐Waals interaction. An ionic interaction appears to be unlikely for this dimer of two cations.

**Figure 5:**
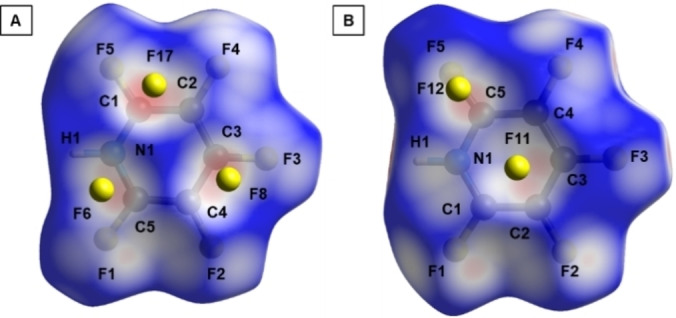
Hirshfeld surface of **2** in the solid state structure of **2** **a**. A – bottom view; B – top view. Red color indicates areas where close‐contact interactions are observed.

The Hirshfeld surface analysis (Figure 5) reveals anion‐π interactions as short contacts between F17‐C2 of 303.58(22) pm, F11‐C2 of 302.9(2) pm, F11‐C3 of 301.5(2) pm and F12‐C5 of 294.06(19) pm (Figure 5). The shortest contact is found for F17‐C1 with a distance of 282.32(19) pm.

**Figure 6:**
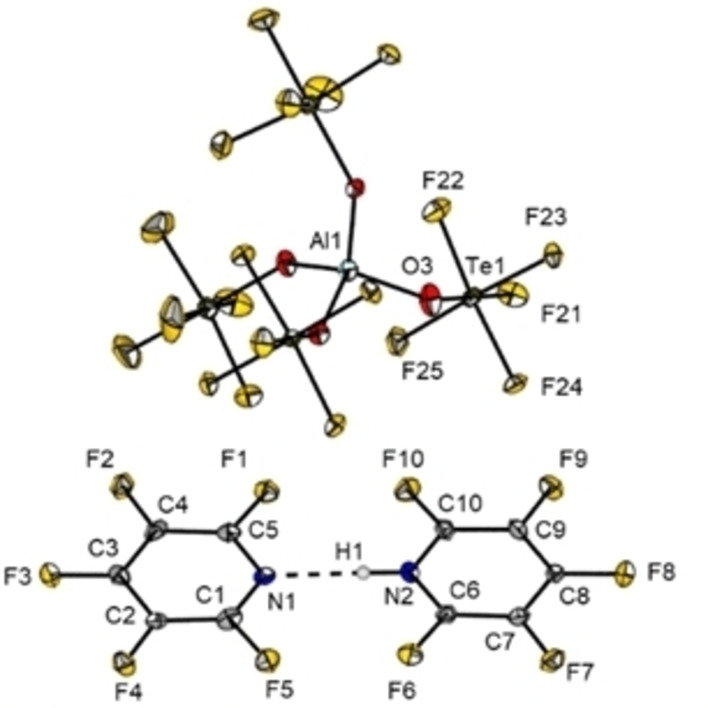
Molecular structure of **5** **a** in the solid state.

From the reaction of **1** **a** with an excess of C_5_F_5_N the salt [(C_5_F_5_N)_2_H][Al(OTeF_5_)_4_] (**5** **a**) was obtained. It crystallizes in the monoclinic space group *Cc* (Figure 6). In the solid state, a pentafluoropyridinum cation interacts with its N−H moiety with the nitrogen atom of another pentafluoropyridine forming a pyridinium‐pyridine dimer, as it has been described for the non‐fluorinated analogue before.^[11]^

**Figure 7:**
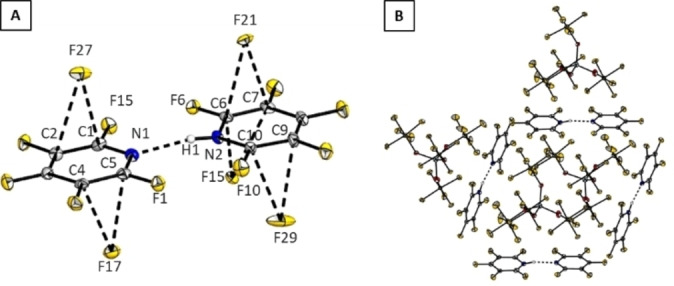
Crystal packing of **5** **a** (B) with a closer view on the dimeric cation **5** (A). Thermal ellipsoids are shown at 50 % probability level. Selected bond lengths [pm]: N1*‐*N2 275.46(83), F5*‐*F6 270.71(79), F1*‐*F10 267.12(83), F27*‐*C2 310.65(97), F27*‐*C1 304.33(100), F17*‐*C4 316.4(1), F17*‐*C5 301.53(98), F21*‐*C6 305.02(97), F21*‐*C7 312.37(99), F15*‐*C6 296.91(92), F15*‐*C7 308.19(85), F29*‐*C10 314.41(116), F29*‐*C9 301.28(112).

The N−N distance in **5** **a** is measured to be 275.46(83) pm which is about 35 pm shorter than the sum of the van der Waals radii of 310 pm.^[10]^ The pentafluoropyridine rings are arranged co‐planar, which is rather unusual. Single molecule calculations at the B3LYP‐D3/def2‐TZVPP level of theory for the dimer predict a structure with a dihedral angle of about 55.5° to be lower in enegy by 16.4 kJ/mol and find the coplanar arrangement to be a transition state. This can be explained by solid state anion‐π interactions, in which the fluorine atoms of the OTeF_5_ moiety above and below the plane of the pentafluoropyridines are stabilizing the planar arrangement (Figure 7).

The Hirshfeld surface plot depicts short contacts from F27, F21, F17 and F29 of the WCA to the rings of cation **5** (see Supporting Information Figure SI 3.1). For 4‐halotetrafluoropyridines, the halogen bond donor strength weakens in the order I, Br, Cl.^[12]^ The halogen bond donor strength can be increased considerably by N‐methylation, like it was shown in case of 4‐iodo‐2,3,5,6‐tetrafluoropyridine and resulted in the formation of a halogen bond with the WCA [Al(OTeF_5_)_4_]^−^ in the solid state.^[13]^ Analogously, an increase of the halogen bond donor strength in halogenated pyridines should be achieved by their protonation. In order to study this behaviour, we reacted one equivalent of 4‐chloro‐2,3,5,6‐tetrafluoropyridine with **1** **a** (Scheme 1).

**Figure 8:**
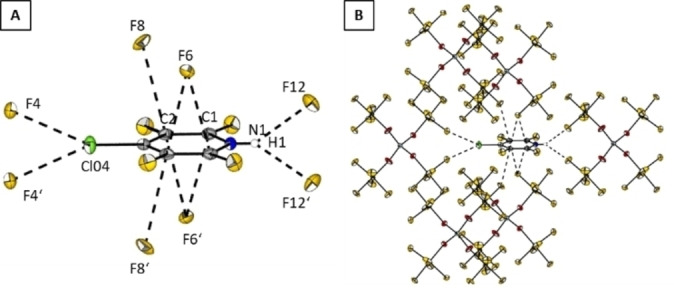
Crystal packing of **3** **a** (B) with a closer view on weak interactions of the cation **3** (A). Thermal ellipsoids are shown at 50 % probability level. Dashed lines show short contacts [pm]: C1‐F6 289.9(29), C2‐F6 286.2(29), C2‐F8 298.3(26), Cl4‐F4 302.0(17).

[C_5_F_4_ClNH][Al(OTeF_5_)_4_] (**3** **a)** crystallizes in the monoclinic space group *C*2/*c* (Figure 8) by slowly cooling the reaction mixture to −24 °C.

**Figure 9:**
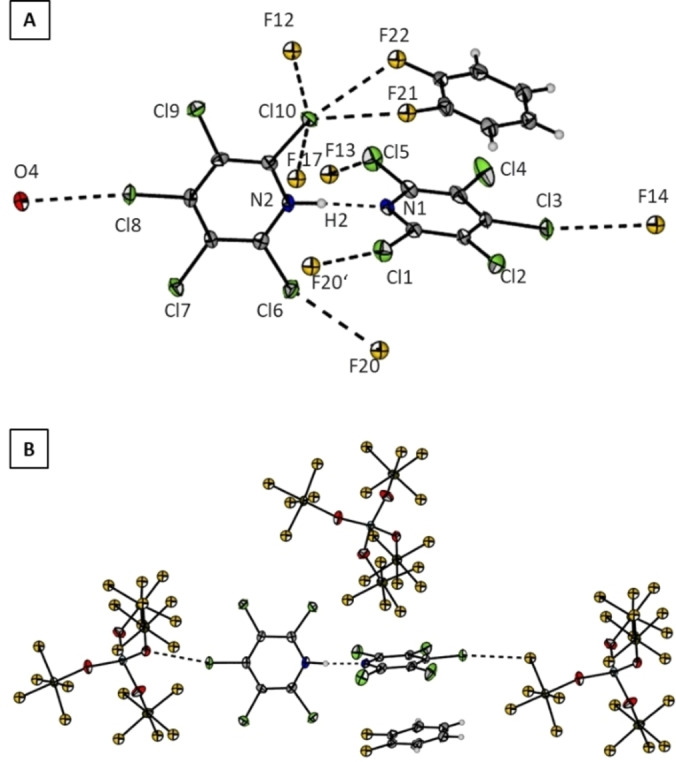
Crystal packing of **6** **a**⋅C_6_H_4_F_2_ (B) with a closer view on the cation **6** (A).Thermal ellipsoids are shown at 50 % probability level. Dashed lines stand for short contacts [pm]: O4*‐*Cl8 301.71(18), Cl10*‐*F12 311.01(18), Cl10*‐*F22 307.43(19), Cl10*‐*F21 300.34(19), Cl10*‐*F17 311.59(17), Cl5*‐*F13 317.93(19), N1*‐*N2 274.46(29), Cl1*‐*F20’ 312.06(18), Cl6*‐*F20 307.47(18), Cl3*‐*F14 312.3(2).

The N−H moiety is interacting with two fluorine atoms, F12 and F12’, via close fluorine‐specific hydrogen bond interactions. A similar arrangement is found for the chlorine atom Cl4 and the fluorine atoms F4 and F4’, respectively. The rather strong halogen‐halogen bond exhibts a chlorine‐fluorine distance of 302.0(17) pm which is 20 pm below the sum of the van der Waals radii. Multiple short F_Anion_‐C_Ar_ contacts below the sum of the van der Waals radii of 317 pm were found.^[10]^ For the anion‐π interaction in heteroaromatic systems, the α‐carbon next to the heteroatom is usually found to form the strongest interactions.^[6]^ This is also the case for **3** **a** in the solid state. Hirshfeld surface analysis reveals interactions between the fluorine atoms F8 and F6 of the WCA and the π‐system of cation **3** (Figure S3.2). The reaction of two equivalents of pentachloropyridine with **1** **a** yielded single crystals of [(C_5_Cl_5_N)_2_H][Al(OTeF_5_)_4_]⋅C_6_H_4_F_2_ (**6** **a**⋅C_6_H_4_F_2_). The compound crystallizes in the monoclinic space group *P*2_1_/*c* (Figure 9).

As for the fluorine analogue **5** **a** the pyridine rings form a pyridinium‐pyridine dimer. The N−N distances in dimers **5** **a** and **6** **a** are indistinguishable within the experimental error. Moreover, the two pentachloropyridine rings in **6** are staggered with an angle between the planes of 95.8(1)°, in contrast to the fluorinated analogue **5** which is arranged coplanar (Figure 4). Single molecule calculations on B3LYP‐D3/def2‐TZVPP level of theory of the perchlorinated dimer **6** find the coplanar arrangement to be a 2^nd^ order saddle point that is 68.2 kJ/mol higher in energy than the staggered conformation with a dihedral angle of 90.2° representing the global minium. This structural difference in the solid state between the cations **5** and **6** can be attributed to the weaker anion‐π interactions (see Figure 2) as well as the bigger repulsion between the chlorine atoms in 2,6‐position. One of the perchloropyridine rings shows a π‐interaction with a cocrystalized *o*DFB molecule. They exhibit a face‐to‐face arrangement with a centroid‐centroid distance of 355.8(1) pm which indicates a rather strong interaction.^[14]^ Several anion‐π interactions are observed between the fluorine atoms F1, F9, F16, and F19 of the WCA and **6** (see Supporting Information Figure S3.3). Additionally, a number of halogen interactions are observed beween the chlorine atoms in 2,6‐ and 4‐positions of **6** and a) fluorine atoms (d(Cl6‐F20)=307.47(18) pm, d(Cl3‐F14)=312.3(2) pm, d(Cl15‐F13)=317.93(19) pm), b) oxygen atoms (d(Cl8‐O4)=301.71(18) pm) of the WCA, and c) fluorine atoms of the co‐crystalized *o*DFB (d(Cl10‐F22)=307.43(19) pm, d(Cl10‐F21)=300.34(19) pm).

In the IR spectra of **2** **a** and **3** **a** several broad bands for the N−H stretch are observed. The N−H band for **2** **a** exhibits two maxima at 3104 cm^−1^ and 2953 cm^−1^, respectively. The N−H band for **3** **a** shows three maxima at 3301 cm^−1^, 3204 cm^−1^ and 2953 cm^−1^, respectively. In contrast to the spectra of the monomeric cations the spectra of the pyridinum‐pyridine dimers show very broad bands in the region from 3000 to 1750 cm^−1^ for **5** **a** and from 3500 to 2000 cm^−1^ for **6** **a** attributed to the N−H stretching vibration. This is well combarable to the reported spectrum of the nonhalogenated pyridinum‐pyridine dimer.^[15]^

## Conclusion

The Brønsted superacid [*o*‐C_6_H_4_F_2_‐H][Al(OTeF_5_)_4_] is able to protonate a series of the very weakly basic perhalogenated pyridines. The cations [C_5_F_5_NH]^+^, [C_5_F_4_ClNH]^+^, [(C_5_Cl_5_N)_2_H]^+^, and [(C_5_F_5_N)_2_H]^+^ were structurally characterized for the first time. In the solid state rather strong noncovalent interactions between these electron deficient aromatic cations and the weakly coordinating anion [Al(OTeF_5_)_4_]^−^ are observed. Due to the protonation of the halogenated pyridines, their ability to form halogen bonds as well as anion‐π interactions is increased compared to the neutral species.

## Experimental Section

For detailed experimental data, see electronic Supporting Information. All reactions were carried out under inert conditions using standard Schlenk techniques. Glass vessels were greased with Triboflon III. All solid materials and triethylaluminium (Al(C_2_H_5_)_3_, 93 %) were handled inside a glove box with an atmosphere of dry argon (O_2_<0.5 ppm, H_2_O<0.5 ppm). The pentafluoroorthotelluric acid was synthesized as reported in the literature.^[16]^ All solvents were dried either with CaH_2_ or with Sicapent® before use. IR spectra were collected on a Bruker ALPHA FTIR spectrometer equipped with a diamond ATR attachment in an argon‐filled glove box. NMR spectra were recorded on either a JEOL 400 MHz ECS or ECZ‐R spectrometer. Reported chemical shifts are referenced to the Ξ values given in IUPAC recommendations of 2008 using the ^2^H signal of the deuterated solvent as internal reference. For external locking [D_6_]‐acetone was flame sealed in a glass capillary and the lock oscillator frequency was adjusted to give *δ*(^1^H)=7.26 ppm for a CHCl_3_ sample locked on the capillary. Chemical shifts and coupling constants of strongly coupled spin systems are given as simulated in *g*NMR.^[17]^ Crystal structures were obtained on a Bruker D_8_ Venture diffractometer with a PHOTON 100 CMOS area detector using Mo−Kα radiation. Single crystals were coated with a perfluoroether oil at −25 °C and selected under nitrogen atmosphere. Using Olex2,^[18]^ the structures were solved with the ShelXT^[19]^ structure solution program by intrinsic phasing and refined with the ShelXL^[20]^ refinement package using least‐squares minimization.

## CCDC

Deposition Number (s) 2082701 (for **2** **a**), 2074488 (for **5** **a**), 2082692 (for **3** **a**), 2074489 (for **6** **a**) contain(s) the supplementary crystallographic data for this paper. These data are provided free of charge by the joint Cambridge Crystallographic Data Centre and Fachinformationszentrum Karlsruhe Access Structures service.

All calculations were performed using a general‐purpose High‐Performance Computer at ZEDAT (CURTA),^[21]^ provided by Freie Universität Berlin. For density functional calculations the Gaussian 16^[22]^ software was used with its implementation of B3LYP, and Grimme‐D3^[23]^ together with the basis set def2‐TZVPP.^[24]^ Calculated structures, as well as crystal structures, were visualized with Diamond.^[25]^ Electrostatic potentials were calculated using the Turbomole software and visualized with a VMD editor. Molecular Hirshfeld surface contours were performed using the Crystal Explorer. In this study, all the Hirshfeld surfaces were generated using a high (standard) surface resolution. For **5** **a**, the 3‐D dnorm surfaces were mapped using a fixed color scale of −0.4727 (red) to 0.5303 (blue), using an isovalue of 0.5. For **2** **a**, **3** **a** and **6** **a**⋅*o*‐C_6_H_4_F_2_, the 3‐D dnorm surfaces were mapped using a fixed color scale of −0.0964 (red) to 0.9305 (blue), using an isovalue of 0.5. Red contours indicated a contact less than the sum of the van der Waals radii of the respective elements. Blue and white contours indicate that the nearest external atom is at a distance greater or equal to the sum of the van der Waals radii respectively from atomic coordinates. Atoms in Molecules (AIM) analysis of an isolated [C_5_F_5_NH]^+^‐dimer based on calculations at B3LYP‐D3/cc‐pVTZ^[26]^ level were performed with the MultiWFN program.^[27]^ The dimer structure was extracted from the experimental crystal structure and not further optimized.

**Synthesis of [C_5_F_5_NH][Al(OTeF_5_)_4_] (2** **a)**: A sample of Al(C_2_H_5_)_3_ (78 mg, 0.68 mmol, 1 equiv.) was dissolved in 3 mL of *ortho*‐difluorobenzene (*o*DFB). The solution was degassed and HOTeF_5_ (652 mg, 2.72 mmol, 4 equiv.) was condensed onto it at −196 °C. The reaction mixture was warmed up to −30 °C. Afterwards a gas bubbler was added and the reaction mixture was stirred for 30 min. A yellow solution was obtained indicating the formation of the corresponding Brønsted superacid. C_5_F_5_N (115 mg, 0.68 mmol, 1 equiv.) was condensed into a Schlenk tube with a PTFE cap and dissolved in 2 mL of *o*DFB. This solution was added to reaction vessel via syringe under an argon stream. The gas bubbler was exchanged with a stopper and the mixture was stirred for another 30 min at −30 °C. The pressure in the reaction vessel was reduced and the flask was placed in a −24 °C fridge for crystallization. [C_5_F_5_NH][Al(OTeF_5_)_4_] was obtained as colourless crystals. ^**1**^ **H** **NMR** (401 MHz, ext. [D_6_]‐acetone, 22 °C): *δ*=12.1 [s, 1H, N−H] ppm; ^**19**^ **F** **NMR** (377 MHz, ext. [D_6_]‐acetone, 22 °C): *δ*=−40.5 [m, ^1^F_A_, ^*2*^ *J*(^19^F, ^19^F)=187 Hz, ^*1*^ *J*(^19^F_A_, ^125^Te)=3350 Hz], −47.6 [m, 4F_B_, ^*1*^ *J*(^125^Te,^19^F_B_)=3474 Hz], −95.2 [m, 2F_XX’_], −100.1 [m, 1F_Z_], −153.1 [m, 2F_YY’_] ppm; ^**27**^ **Al** **NMR** (104 MHz, ext. [D_6_]‐acetone, 22 °C): δ=49.9 [s, 75 % [Al(OTeF_5_)_4_]^−^, d, 22.5 % [Al(OTeF_5_)_3_(O^125^TeF_5_)]^−^, ^*2*^ *J*(^27^Al,^125^Te)=72 Hz; t, 2.5 % [Al(OTeF_5_)_2_(O^125^TeF_5_)_2_]^−^, ^*2*^ *J*(^27^Al, ^125^Te)=69 Hz] ppm. **IR** (ATR, 22 °C): $\tilde{v}$ =3103 (w), 2953 (w), 1691 (m), 1610 (m), 1535 (w), 1466 (w), 1325 (w), 1122 (m), 988 (w), 927 [s, v_as_(Al−O)], 799 [w, v(CN)], 684 [vs, v_as_(Te−F_4_)], 606 [w, v_as_(O−Te−F)], 553 [m, vs(Al−O)] cm^−1^. **Crystal Data for [C_5_F_5_NH][Al(OTeF_5_)_4_]** (M=1151.45 g/mol): triclinic, space group *P‐1* (no. 2), a=9.4813(3) Å, b=10.9228(5) Å, c=12.0967(6) Å, α=78.920(2)°, β=67.723(2)°, γ=86.922(2)°, V=1137.40(9) Å^3^, Z=2, T=100.0 K, μ(MoKα)=5.339 mm^−1^, D_calc_=3.362 g/cm^3^, 109127 reflections measured (3.704°≤2Θ≤56.66°), 5673 unique (R_int_=0.0405, R_sigma_=0.0146) which were used in all calculations. The final R_1_ was 0.0141 (I >2σ(I)) and *w*R_2_ was 0.0313 (all data).

**Synthesis of [C_5_F_4_ClNH][Al(OTeF_5_)_4_] (3** **a)**: The synthesis was analogous to the one of [C_5_F_5_NH][Al(OTeF_5_)_4_] using C_5_F_4_ClN (130 mg, 0.7 mmol, 1 equiv.) that was previously dissolved in 2 mL of *o*DFB. After the reaction was complete, all volatile parts were removed in vacuo. The flask with the remaining solution was put into a −24 °C fridge for crystallization. [C_5_F_4_ClNH][Al(OTeF_5_)_4_] was obtained as colourless crystals. ^**1**^ **H** **NMR** (401 MHz, ext. [D_6_]‐acetone, 22 °C): *δ*=12.2 [s, 1H, N−H] ppm. ^**19**^ **F** **NMR** (377 MHz, ext. [D_6_]‐acetone, 22 °C): *δ=*−38.8 [m, 1F_A_, ^2^ *J*(^19^F_A_,^19^F_B_)=188, ^1^ *J*(^19^F_A_,^125^Te)=3353 Hz], −45.7 [m, 4F_B_, ^1^ *J*(^125^Te,^19^F_B_)=3473 Hz], −99.1 [m, 2F_XX’_], −133.3 [m, 2F_YY’_] ppm. ^**27**^ **Al** **NMR** (104 MHz, ext. [D_6_]‐acetone, 22 °C): *δ=*50.1 [s, 75 %, [Al(OTeF_5_)_4_]^−^; d, 22.5 % [Al(OTeF_5_)_3_(O^125^TeF_5_)]^−^, ^2^ *J*(^27^Al,^125^Te)=72 Hz; t, 2.5 % [Al(OTeF_5_)_2_(O^125^TeF_5_)_2_]^−^, ^2^ *J*(^27^Al,^125^Te)=73 Hz] ppm. **IR** (ATR, 22 °C): $\tilde{v}$ =3301 (w), 3204 (m), 2953 (vw), 1671 (w), 1649 (w), 1570 (m), 1471 (w), 1445 (w), 1305 (m), 928 [vs, *v_as_* (Al−O)], 687 [vs, *v_as_* (Te−F_4_)], 636 [m, *v_as_* (O−Te−F)], 633 (m), 618 (m), 553 [m, vs(Al−O)], 246 (w) cm^−1^. **Crystal Data for [C_5_F_4_ClNH][Al(OTeF_5_)_4_]** (M=1167.90 g/mol): monoclinic, space group *C*2/*c* (no. 15), a=13.0024(5) Å, b=16.6373(7) Å, c=11.4609(5) Å, β=106.524(2)°, V=2376.88(17) Å^3^, Z=4, T=100.0 K, μ(MoKα)=5.215 mm^−1^, D_calc_=3.264 g/cm^3^, 40800 reflections measured (4.082≤2Θ≤56.74°), 2979 unique (R_int_=0.0328, R_sigma_=0.0141) which were used in all calculations. The final R_1_ was 0.0163 (I>2σ(I)) and *w*R_2_ was 0.0337 (all data).

**Synthesis of [(C_5_F_5_N)_2_H][Al(OTeF**_***5***_ **)**_***4***_ **] (5** **a)**: The synthesis was analogous to the one of [C_5_F_5_NH][Al(OTeF_5_)_4_] using 10 equivalents of C_5_F_5_N (1180 mg, 7.0 mmol, 10 equiv.) that were previously dissolved in 3 mL of *o*DFB. [(C_5_F_5_N)_2_H][Al(OTeF_5_)_4_] was obtained as a colourless crystals. ^**1**^ **H** **NMR** (401 MHz, ext. [D_6_]‐acetone, 22 °C): δ=11.5 [s, 1H, N−H] ppm; ^**19**^ **F** **NMR** (377 MHz, ext. [D_6_]‐acetone, 22 °C): δ=−38.7 [m, 1F_A_, ^*2*^ *J*(^19^F, ^19^F)=193 Hz, ^*1*^ *J*(^19^F_A_,^125^Te)=3363 Hz], −45.8 [m, 4F_B_, ^*1*^ *J*(^125^Te,^19^F_B_)=3414 Hz], −89.7 [m, 2F_XX’_], −132.2 [m, 2F_Z_], −161.8 [m, 2F_YY’_] ppm; ^**27**^ **Al** **NMR** (104 MHz, ext. [D_6_]‐acetone, 22 °C): δ=50.1 [s, 75 % [Al(OTeF_5_)_4_]^−^, d, 22.5 % [Al(OTeF_5_)_3_(O^125^TeF_5_)]^−^, ^*2*^ *J*(^27^Al,^125^Te)=73 Hz; t, 2.5 % [Al(OTeF_5_)_2_(O^125^TeF_5_)_2_]^−^, ^*2*^ *J*(^27^Al, ^125^Te)=73.2 Hz] ppm. **IR** (ATR, 22 °C): $\tilde{v}$ =2323 (w), 1848 (w), 1666 (m), 1610 (m), 1442 (s), 1394 (w), 1307 (m), 1213 (vw), 1104 (m), 1092 (m), 930 [vs, *v_as_* (Al−O)], 685 [vs, *v_as_* (Te−F_4_)], 641 [m, *v_as_* (O−Te−F)], 627 (m), 553 [m, vs(Al−O)], 431 (w) cm^−1^. **Crystal Data for [(C_5_F_5_N)_2_H][Al(OTeF_5_)_4_]** (*M=*1320.51 g/mol): monoclinic, space group *Cc* (no. 9), *a*=17.6950(12) Å, *b*=18.7008(13) Å, *c*=9.0499(6) Å, *β*=107.858(2)°, *V=*2850.4(3) Å^3^, *Z*=4, *T*=100.0 K, μ(MoKα)=4.308 mm^−1^, D_calc_=3.077 g/cm^3^, 136449 reflections measured (4.356°≤2Θ≤66.34°), 10560 unique (*R*_int_=0.0486, R_sigma_=0.0234) which were used in all calculations. The final *R*_1_ was 0.0306 (I>2σ(I)) and *wR*_2_ was 0.0607 (all data).

**Synthesis of [(C_5_Cl_5_N)_2_H][Al(OTeF**_***5***_ **)**_***4***_ **]⋅C_6_H_4_F_2_ (6** **a⋅C_6_H_4_F_2_)**: The synthesis was analogous to the one of [C_5_Cl_5_NH][Al(OTeF_5_)_4_] using 2 equivalents of C_5_Cl_5_N (350 mg, 1.4 mmol, 2 equiv.) that were previously dissolved in 3 mL of *o*DFB. [(C_5_Cl_5_N)_2_H][Al(OTeF_*5*_)_*4*_] was obtained as colourless crystals. ^**1**^ **H** **NMR** (401 MHz, ext. [D_6_]‐acetone, 22 °C): δ=17.5 [s, 1H, N−H] ppm; ^**19**^ **F** **NMR** (377 MHz, ext. [D_6_]‐acetone, 22 °C): δ=−38.6 [m, 1F_A_, ^*2*^ *J*(^19^F, ^19^F)=193 Hz, ^*1*^ *J*(^19^F_A_,^125^Te)=3333 Hz], −45.7 [m, 4F_B_, ^*1*^ *J*(^125^Te,^19^F_B_)=3447 Hz] ppm; ^**27**^ **Al** **NMR** (104 MHz, ext. [D_6_]‐acetone, 22 °C): δ=50.0 [s, 75 % [Al(OTeF_5_)_4_]^−^, d, 22.5 % [Al(OTeF_5_)_3_(O^125^TeF_5_)]^−^, ^*2*^ *J*(^27^Al,^125^Te)=73.0 Hz; t, 2.5 % [Al(OTeF_5_)_2_(O^125^TeF_5_)_2_]^−^, ^*2*^ *J*(^27^Al, ^125^Te)=73.0 Hz] ppm. **IR** (ATR, 22 °C): $\tilde{v}$ =3555 (m), 3001 (vw), 2953 (vw), 1619 (w), 1541 (w), 1491 (w), 1449 (w), 1403 (w), 1143 (m), 1120 (m), 1088 (m), 1085 (m), 1007 (m), 922 [vs, *v_as_* (Al−O)], 859 (m), 693 [vs, *v_as_* (Te−F_4_)], 633 (m), 618 (m), 555 [m, vs(Al−O)], 420 (s) cm^−1^. **Crystal Data for [(C_5_Cl_5_N)_2_H][Al(OTeF_5_)_4_]⋅C_6_H_4_F_2_** (M=1599.10 g/mol): monoclinic, space group *P*21/*c* (no. 14), a=12.978(3) Å, b=22.529(5) Å, c=14.071(3) Å, β=96.167(8)°, V=4090.4(15) Å^3^, Z=4, T=100.1 K, μ(MoKα)=3.632 mm^−1^, D_calc_=2.597 g/cm^3^, 173293 reflections measured (4.442°≤2Θ≤56.616°), 10174 unique (*R*_int_=0.0402, *R*_sigma_=0.0152) which were used in all calculations. The final R_1_ was 0.0208 (I>2σ(I)) and *w*R_2_ was 0.0484 (all data).

## Conflict of interest

The authors declare no conflict of interest.

1

## Supporting information

As a service to our authors and readers, this journal provides supporting information supplied by the authors. Such materials are peer reviewed and may be re‐organized for online delivery, but are not copy‐edited or typeset. Technical support issues arising from supporting information (other than missing files) should be addressed to the authors.

Supporting Information

## Data Availability

The data that support the findings of this study are available from the corresponding author upon reasonable request.
