# Supplementary material for: Noncovalent Interactions in Halogenated Pyridinium Salts of the Weakly Coordinating Anion [Al(OTeF5)4]−
Source: Chemistry. 2022 Dec 8;29(5):e202202749. doi: 10.1002/chem.202202749 (PMC10107151; doi:10.1002/chem.202202749)
Supplement: Supplementary file 1 — Supporting Information [file CHEM-29-0-s001.pdf]

Chemistry–A European Journal

Supporting Information

Noncovalent Interactions in Halogenated Pyridinium Salts of the Weakly Coordinating Anion $[Al(OTeF_5)_4]^-$

S. Kotsyuda, A. N. Toraman, P. Voßnacker, M. A. Ellwanger, S. Steinhauer, C. Müller, and S. Riedel*

Supporting Information

Table of Contents

I. NMR Spectra 2

II. Crystallographic Data Tables 8

III. Hirshfeld Plots 15

IV. IR spectra 16

V. Quantum-chemical calculations 18

VI. References 21

I. NMR Spectra

1.1 NMR spectra of $[C_5F_5NH][Al(OTeF_5)_4]$ (2a)

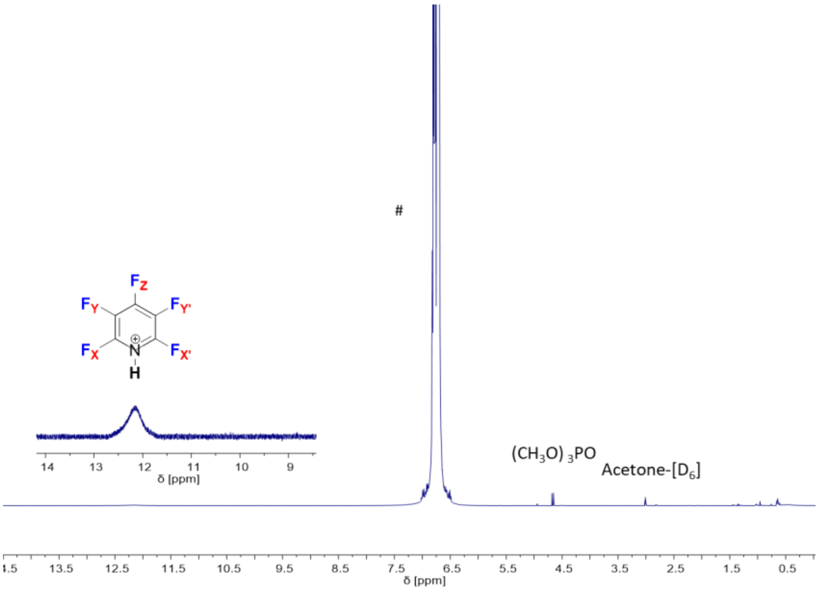

Fig. S.1.1.1. ^{1}H NMR (ext. [D_6]acetone, 25 °C, 401 MHz). Spectrum of $[C_5F_5NH][Al(OTeF_5)_4]$ in 1,2-difluorobenzene (marked as #).

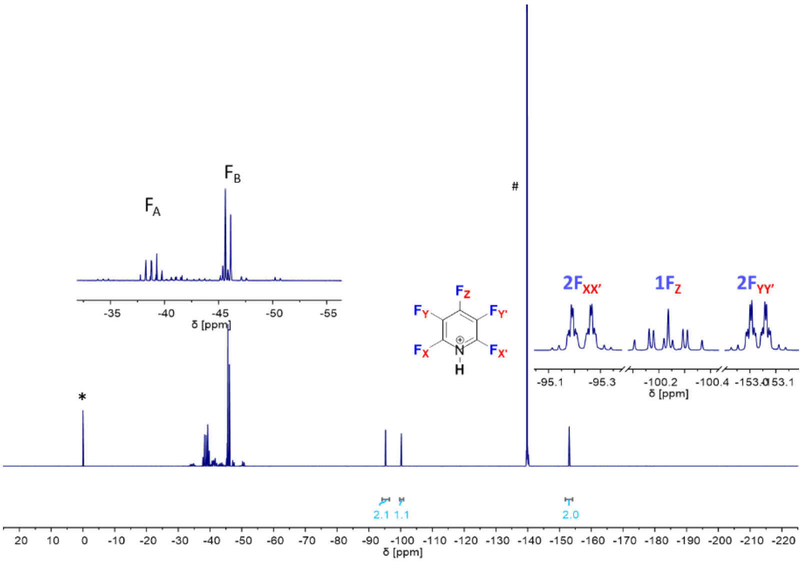

Fig. S.1.1.2 ^{19}F NMR (ext. [D_6]acetone, 25 °C, 377 MHz). Spectrum of $[C_5F_5NH][Al(OTeF_5)_4]$ in 1,2-difluorobenzene. Corresponding resonances are labelled. * – ext. $CFCl_3$; # – 1,2-difluorobenzene; F_A in black– axial fluorine atom of the $–OTeF_5$ moiety, F_B in black – equatorial fluorine atoms of the $–OTeF_5$ moiety.

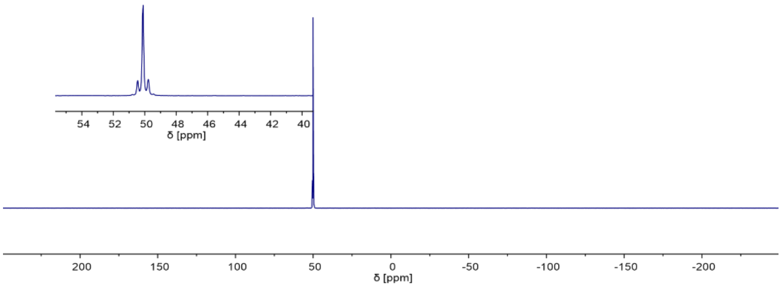

Fig. S.1.1.3. ^{27}Al NMR (ext. [D_6]acetone, 25 °C, 104 MHz). Spectrum of $[C_5F_5NH][Al(OTeF_5)_4]$ in 1,2-difluorobenzene.

1.2 NMR spectra of $[C_5F_4ClNH][Al(OTeF_5)_4]$ (3a)

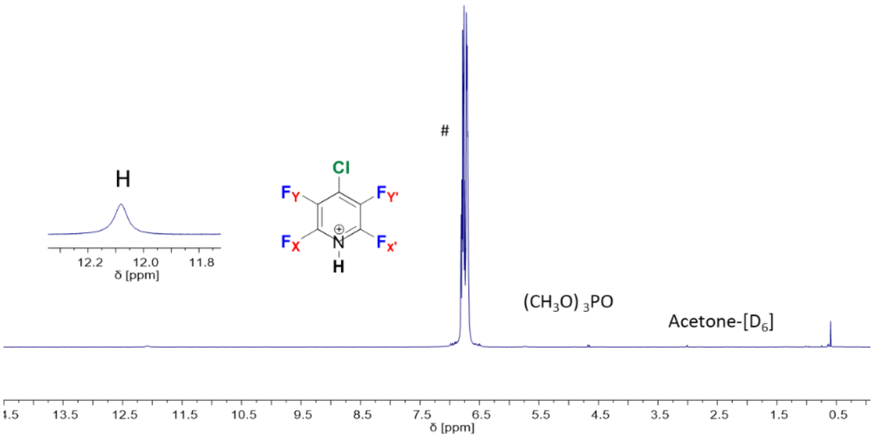

Fig. S.1.2.1. ¹H NMR (ext. [D₆]acetone, 25 °C, 401 MHz). Spectrum of $[C_5F_4ClNH][Al(OTeF_5)_4]$ in 1,2-difluorobenzene (marked as #).

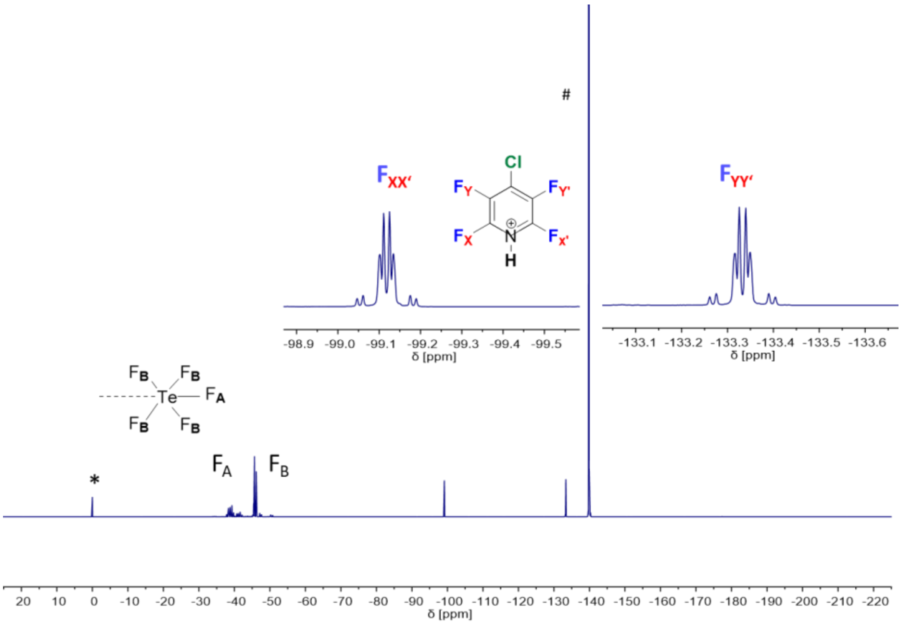

Fig. S.1.2.2. ¹⁹F NMR (ext. [D₆]acetone, 25 °C, 377 MHz). Spectrum of $[C_5F_4ClNH][Al(OTeF_5)_4]$ in 1,2-difluorobenzene. Corresponding resonances are labeled. * – ext. $CFCl_3$; # – 1,2-difluorobenzene; F_A in black– axial fluorine atom of the $–OTeF_5$ moiety, F_B in black – equatorial fluorine atoms of the $–OTeF_5$ moiety.

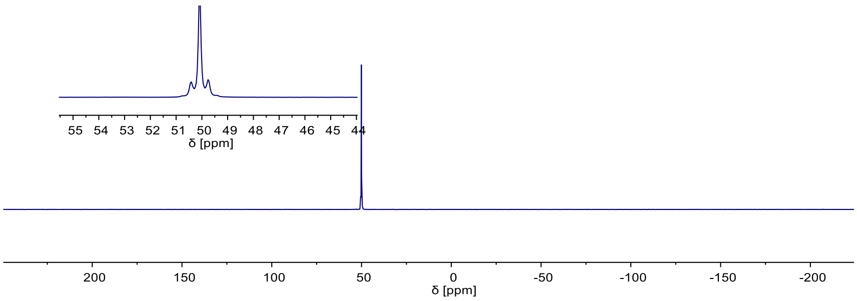

Fig. S.1.2.3. ²⁷Al NMR (ext. [D₆]acetone, 25 °C, 104 MHz). Spectrum of $[C_5F_4ClNH][Al(OTeF_5)_4]$ in 1,2-difluorobenzene.

1.3 NMR spectra of $[(C_5F_5N)_2H][Al(OTeF_5)_4]$ (5a)

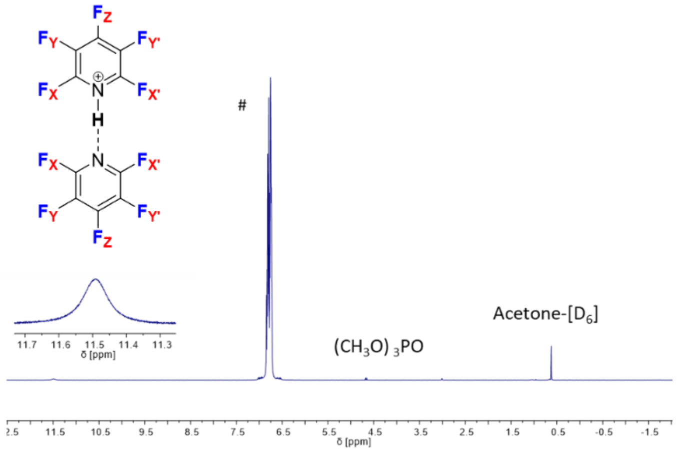

Fig. S.1.3.1. ^{1}H NMR (ext. [D$_6$]acetone, 25 °C, 401 MHz). Spectrum of $[(C_5F_5N)_2H][Al(OTeF_5)_4]$ in 1,2-difluorobenzene (marked as #).

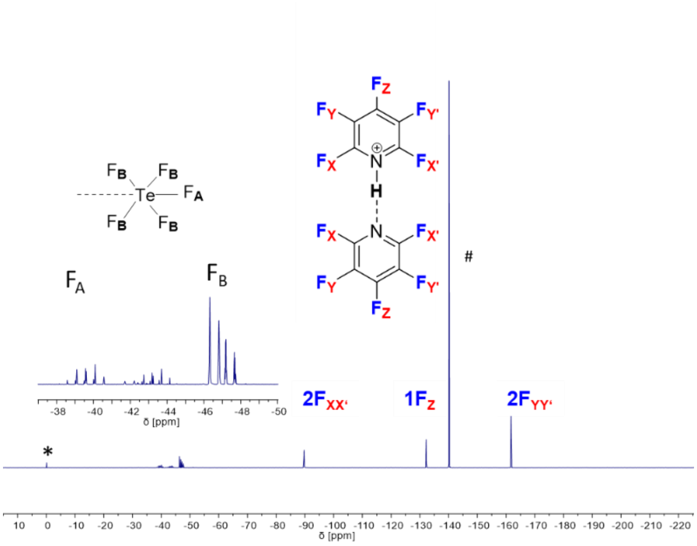

Fig. S.1.3.2. ^{19}F NMR (ext. [D$_6$]acetone, 25 °C, 377 MHz). Spectrum of $[(C_5F_5N)_2H][Al(OTeF_5)_4]$ in 1,2-difluorobenzene. Corresponding resonances are labelled. * – ext. $CFCl_3$; # – 1,2-difluorobenzene; F_A in black– axial fluorine atom of the $–OTeF_5$ moiety, F_B in black – equatorial fluorine atoms of the $–OTeF_5$ moiety.

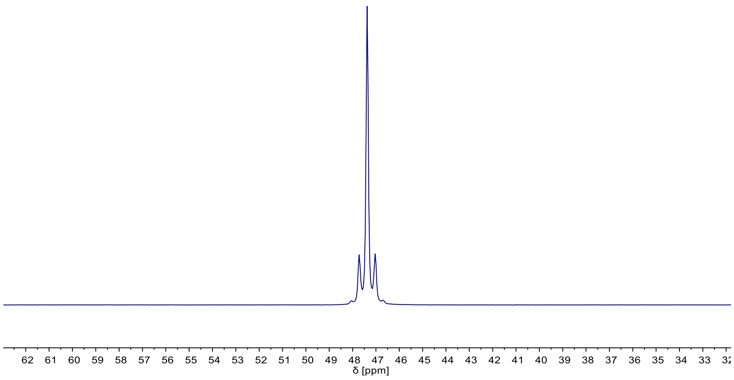

Fig. S.1.3.3. ^{27}Al NMR (ext. [D$_6$]acetone, 25 °C, 104 MHz). Spectrum of $[(C_5F_5N)_2H][Al(OTeF_5)_4]$ in 1,2-difluorobenzene.

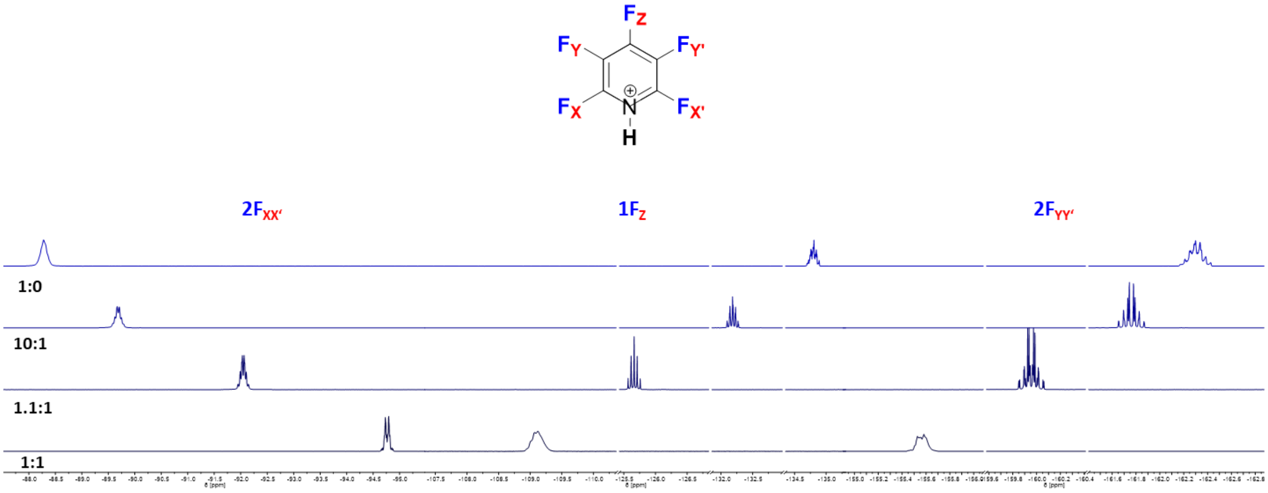

Figure SI 1.3.4. ^{19}F NMR spectrum (ext. [D_6]acetone, 25 °C, 377 MHz) of pentafluoropyridine with different equivalents of [*o*-$C_6H_4F_2$-H][Al($OTeF_5$)$_4$] in 1,2-difluorobenzene.

**Table SI. 1.3.1.** ^{19}F NMR in *o*DFB, δ_F [ppm]				^1H NMR, δ [ppm]
Ratio Py-F_5:[*o*-$C_6H_4F_2$-H][Al($OTeF_5$)$_4$]	**2F$_{XX'}$**	**1F$_Z$**	**2F$_{YY'}$**	**1H**
**1:0** neat py-F_5 in *o*DFB	-88.3	-134.8	-162.3	-
**10:1**	-89.7 **(-1.4)**	-132.2 **(+2.6)**	-161.8 **(+0.5)**	11.5
**1.1:1**	-92.6 **(-4.3)**	-125.6 **(+9.2)**	-160.0 **(+2.3)**	10.5
**1:1**	-94.8 **(-6.5)**	-109.2 **(+25.6)**	-155.5 **(+6.8)**	7.5

**Table SI. 1.3.2.** ^{19}F NMR in SO_2 from lit.[1], δ_F [ppm]			
Ratio Py-F_5:acid	**2F$_{XX'}$**	**1F$_Z$**	**2F$_{YY'}$**
**1:0** neat py-F_5 in SO_2	-89.5	-135.0	-163.5
**1:1** py-F_5 in H_2SO_4	-95.8 **(-6.3)**	-126.0 **(+9.0)**	-160.0 **(+3.5)**
**1:1** py-F_5 in HSO_3F	-97.4 **(-7.9)**	-109.4 **(+25.6)**	-156.7 **(+6.8)**
**1:1** py-F_5 in HSO_3F/SbF_5	-96.4 **(-6.9)**	-102.8 **(+32.2)**	-154.5 **(+7.1)**

1.4 NMR spectra of [(C_5Cl_5N)$_2$H][Al($OTeF_5$)$_4$] (6a)

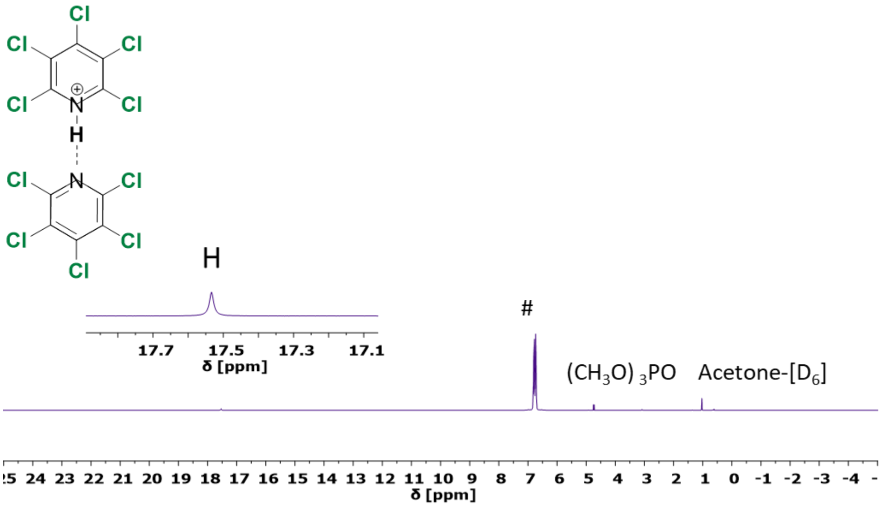

Fig. S.1.4.1. ^{1}H NMR (ext. [D_6]acetone, 25 °C, 401 MHz). Spectrum of [(C_5Cl_5N)$_2$H][Al($OTeF_5$)$_4$] salt in 1,2-difluorobenzene (depicted as #).

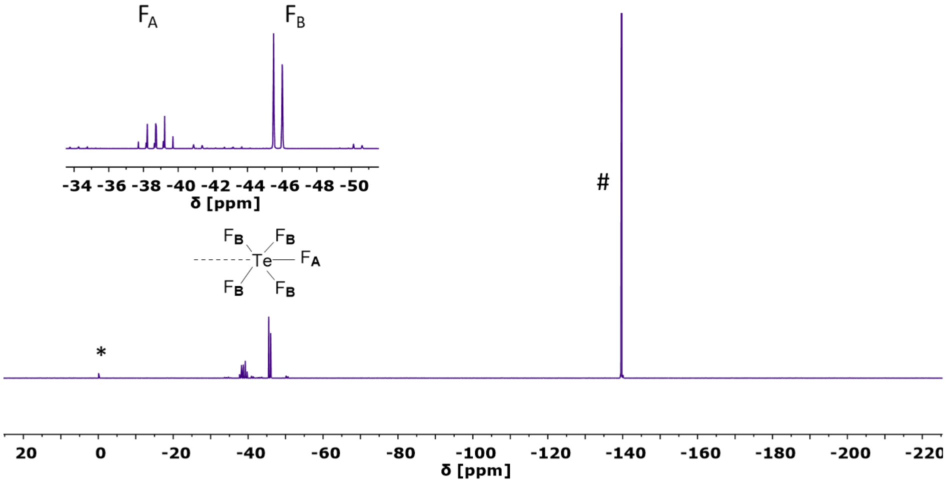

Fig. S.1.4.2. ^{19}F NMR (ext. [D_6]acetone, 25 °C, 377 MHz). Spectrum of [(C_5Cl_5N)$_2$H][Al($OTeF_5$)$_4$] in 1,2-difluorobenzene. Corresponding resonances are labelled. * – ext. $CFCl_3$; # – 1,2-difluorobenzene; F_A– axial fluorine atom of the –$OTeF_5$ moiety, F_B – equatorial fluorine atoms of the –$OTeF_5$ moiety.

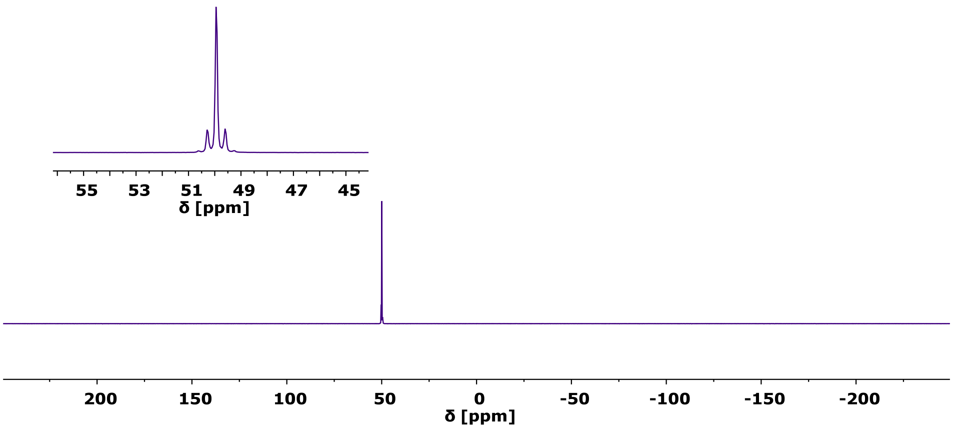

Fig. S.1.4.3. ^{27}Al NMR (ext. [D_6]acetone, 25 °C, 104 MHz). Spectrum of [(C_5Cl_5N)$_2$H][Al($OTeF_5$)$_4$] in 1,2-difluorobenzene.

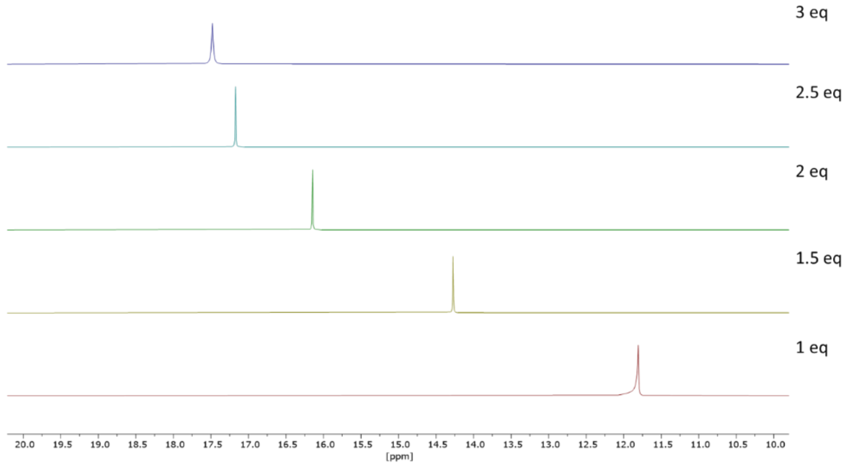

Figure SI 1.4.4. ^{1}H NMR spectrum (ext. [D$_6$]acetone, 25 °C, 401 MHz) of the reaction of 1 equivalent of the Brønsted superacid [*o*-$C_6H_4F_2$-H][Al(OTeF$_5$)$_4$] with different equivalents of C_5Cl_5N in *ortho*-difluorobenzene.

In the following, we treated the Brønsted superacid [*o*-$C_6H_4F_2$-H][Al(OTeF$_5$)$_4$] in *o*DFB with different amounts of C_5Cl_5N in order to investigate the change in the ^{1}H chemical shift. The addition of more than 3 equivalents of C_5Cl_5N to 1 equivalent of [*o*-$C_6H_4F_2$-H][Al(OTeF$_5$)$_4$] did not result in any further change in the chemical shift. The variation of the chemical shifts is presented in Fig. SI 1.4.4. and table SI 1.4.1. For 1 eq. of C_5Cl_5N, a signal is observed at 12 ppm and shifts to lower fields with higher concentrations of C_5Cl_5N, reaching 17.5 ppm, when the molar ratio of C_5Cl_5N is 3 eq.

**Table SI. 1.4.1.** ^{1}H NMR, δ [ppm]	
**Ratio** py-Cl$_5$:[*o*-$C_6H_4F_2$-H][Al(OTeF$_5$)$_4$]	**1H**
**3:1**	17.5
**2.5:1**	17.0
**2:1**	16.0
**1.5:1**	14.5
**1:1**	12.0

II. Crystallographic Data Tables

Table SI 2.1.1. Bond Lengths for $[(C_5F_5N)_2H][Al(OTeF_5)_4]$ (**5a**).

Atom	Atom	Length/Å	Atom	Atom	Length/Å
Te01	O3	1.792(6)	O3	Al1	1.764(6)
Te01	F22	1.839(6)	F6	C6	1.298(9)
Te01	F25	1.839(6)	F10	C10	1.303(9)
Te01	F24	1.848(7)	F2	C4	1.335(8)
Te01	F23	1.843(5)	F3	C3	1.309(8)
Te01	F21	1.843(6)	Al1	O4	1.763(7)
Te02	F11	1.819(6)	Al1	O2	1.754(6)
Te02	F12	1.856(6)	Al1	O1	1.767(7)
Te02	F15	1.848(3)	F8	C8	1.323(8)
Te02	F13	1.824(3)	C9	C8	1.387(11)
Te02	F14	1.835(7)	C9	C10	1.387(10)
Te02	O1	1.821(6)	F7	C7	1.323(8)
Te03	F28	1.818(8)	C5	N1	1.320(10)
Te03	F29	1.825(7)	C5	F1	1.333(9)
Te03	O4	1.804(4)	C5	C4	1.381(10)
Te03	F26	1.838(4)	F5	C1	1.314(9)
Te03	F27	1.847(7)	N1	C1	1.336(10)
Te03	F30	1.835(7)	C8	C7	1.369(11)
Te04	F20	1.825(5)	C10	N2	1.331(9)
Te04	F17	1.843(7)	N2	C6	1.337(9)
Te04	F16	1.831(8)	C7	C6	1.375(10)
Te04	F18	1.852(6)	C4	C3	1.389(11)
Te04	O2	1.810(7)	F4	C2	1.328(8)
Te04	F19	1.860(6)	C2	C3	1.374(10)
F9	C9	1.328(9)	C2	C1	1.380(10)

Table SI 2.2.2. Bond Angles for $[(C_5F_5N)_2H][Al(OTeF_5)_4]$ (**5a**).

Atom	Atom	Atom	Angle/˚	Atom	Atom	Atom	Angle/˚	Atom	Atom	Atom	Angle/˚	Atom	Atom	Atom	Angle/˚
O3	Te01	F22	94.7(3)	F16	Te04	F17	85.8(3)	F29	Te03	F26	86.1(5)	F2	C4	C5	120.3(7)
O3	Te01	F25	93.3(3)	F16	Te04	F18	86.3(3)	F29	Te03	F27	172.7(2)	F2	C4	C3	121.4(7)
O3	Te01	F24	93.8(3)	F16	Te04	F19	86.1(3)	F29	Te03	F30	90.7(4)	C5	C4	C3	118.3(7)
O3	Te01	F23	93.6(3)	F18	Te04	F19	91.6(3)	O4	Te03	F28	93.4(4)	Al1	O2	Te04	137.0(4)
O3	Te01	F21	178.9(3)	O2	Te04	F20	93.1(3)	O4	Te03	F29	94.4(4)	Al1	O1	Te02	138.9(4)
F22	Te01	F24	171.5(3)	O2	Te04	F17	94.8(3)	O4	Te03	F26	178.5(5)	F4	C2	C3	121.5(7)
F22	Te01	F23	89.9(2)	O2	Te04	F16	179.3(4)	O4	Te03	F27	93.0(4)	F4	C2	C1	120.0(7)
F22	Te01	F21	84.8(3)	O2	Te04	F18	94.1(3)	O4	Te03	F30	94.0(4)	C3	C2	C1	118.5(7)
F25	Te01	F22	91.5(3)	O2	Te04	F19	93.3(3)	F26	Te03	F27	86.5(5)	F3	C3	C4	120.4(7)
F25	Te01	F24	89.1(3)	Al1	O3	Te01	133.3(4)	F30	Te03	F26	87.5(4)	F3	C3	C2	120.2(7)
F25	Te01	F23	172.9(2)	O3	Al1	O1	106.7(3)	F30	Te03	F27	89.2(3)	C2	C3	C4	119.3(7)
F25	Te01	F21	85.7(3)	O4	Al1	O3	110.8(4)	F20	Te04	F17	87.9(3)	F6	C6	N2	116.9(6)
F23	Te01	F24	88.5(2)	O4	Al1	O1	109.8(3)	F20	Te04	F16	86.5(3)	F6	C6	C7	120.3(7)
F21	Te01	F24	86.8(3)	O2	Al1	O3	111.6(3)	F20	Te04	F18	171.9(3)	N2	C6	C7	122.7(7)
F21	Te01	F23	87.4(2)	O2	Al1	O4	108.4(4)	F20	Te04	F19	91.6(3)	F5	C1	N1	116.7(7)
F11	Te02	F12	89.6(3)	O2	Al1	O1	109.5(3)	F17	Te04	F18	87.9(3)	F5	C1	C2	121.1(7)
F11	Te02	F15	86.7(3)	Al1	O4	Te03	137.7(5)	F17	Te04	F19	171.8(3)	N1	C1	C2	122.1(7)
F11	Te02	F13	88.9(4)	F9	C9	C8	121.4(7)								
F11	Te02	F14	87.3(3)	F9	C9	C10	120.4(7)								
F11	Te02	O1	178.6(2)	C8	C9	C10	118.2(7)								
F15	Te02	F12	89.0(3)	N1	C5	F1	116.8(7)								
F13	Te02	F12	92.1(4)	N1	C5	C4	122.3(7)								
F13	Te02	F15	175.4(4)	F1	C5	C4	120.9(7)								
F13	Te02	F14	91.3(4)	C5	N1	C1	119.4(6)								
F14	Te02	F12	175.3(3)	F8	C8	C9	119.9(7)								
F14	Te02	F15	87.3(3)	F8	C8	C7	120.3(7)								
O1	Te02	F12	91.1(3)	C7	C8	C9	119.8(6)								
O1	Te02	F15	92.1(3)	F10	C10	C9	120.6(7)								
O1	Te02	F13	92.4(4)	F10	C10	N2	117.3(6)								
O1	Te02	F14	91.9(3)	N2	C10	C9	122.0(7)								
F28	Te03	F29	89.4(4)	C10	N2	C6	118.8(6)								
F28	Te03	F26	85.1(4)	F7	C7	C8	121.4(7)								
F28	Te03	F27	89.7(4)	F7	C7	C6	120.3(7)								
F28	Te03	F30	172.55(19)	C8	C7	C6	118.3(7)								

Table SI 2.3.1. Bond Lengths for [C_5F_5NH][$Al(OTeF_5)_4$] (**2a**).

Atom	Atom	Length/Å	Atom	Atom	Length/Å
Te1	F8	1.8358(11)	Te4	F22	1.8294(12)
Te1	F9	1.8310(11)	Te4	F24	1.8260(12)
Te1	F7	1.8353(11)	Te4	F21	1.8263(12)
Te1	F6	1.8409(11)	Te4	O4	1.8062(13)
Te1	F10	1.8321(12)	Al1	O2	1.7441(14)
Te1	O1	1.8128(13)	Al1	O3	1.7423(13)
Te3	F20	1.8280(12)	Al1	O4	1.7441(14)
Te3	F17	1.8425(11)	Al1	O1	1.7445(14)
Te3	F18	1.8381(12)	F3	C3	1.308(2)
Te3	F19	1.8347(11)	F2	C2	1.320(2)
Te3	F16	1.8277(11)	F1	C1	1.300(2)
Te3	O3	1.8152(13)	F5	C5	1.300(2)
Te2	F15	1.8415(11)	F4	C4	1.318(2)
Te2	F12	1.8332(12)	N1	C1	1.334(3)
Te2	F11	1.8411(12)	N1	C5	1.341(3)
Te2	F13	1.8316(12)	C1	C2	1.372(3)
Te2	F14	1.8254(12)	C4	C3	1.384(3)
Te2	O2	1.8123(13)	C4	C5	1.368(3)
Te4	F25	1.8635(12)	C2	C3	1.383(3)
Te4	F23	1.8267(12)			

Table SI 2.3.2. Bond Angles for [C_5F_5NH][$Al(OTeF_5)_4$] (**2a**).

Atom	Atom	Atom	Angle/˚	Atom	Atom	Atom	Angle/˚	Atom	Atom	Atom	Angle/˚
F8	Te1	F6	87.44(5)	O2	Te2	F13	94.85(6)	F13	Te2	F12	90.55(6)
F9	Te1	F8	88.73(6)	O2	Te2	F14	93.70(6)	F13	Te2	F11	87.03(6)
F9	Te1	F7	172.94(5)	F23	Te4	F25	174.21(6)	F14	Te2	F15	89.45(6)
F9	Te1	F6	87.61(5)	F23	Te4	F22	91.16(6)	F14	Te2	F12	172.52(6)
F9	Te1	F10	90.26(6)	F22	Te4	F25	88.72(6)	F14	Te2	F11	87.12(6)
F7	Te1	F8	89.94(6)	F24	Te4	F25	88.17(6)	F14	Te2	F13	91.11(6)
F7	Te1	F6	85.41(5)	F24	Te4	F23	91.30(6)	O2	Te2	F15	92.41(6)
F10	Te1	F8	174.14(5)	F24	Te4	F22	172.91(6)	O2	Te2	F12	93.42(6)
F10	Te1	F7	90.36(6)	F24	Te4	F21	86.90(6)	O2	Te2	F11	177.93(6)
F10	Te1	F6	86.75(6)	F21	Te4	F25	85.98(6)	F2	C2	C1	120.79(17)
O1	Te1	F8	91.29(6)	F21	Te4	F23	88.23(6)	F2	C2	C3	121.06(17)
O1	Te1	F9	93.57(6)	F21	Te4	F22	86.53(6)	C1	C2	C3	118.15(18)
O1	Te1	F7	93.40(6)	O4	Te4	F25	91.05(6)	F3	C3	C4	119.58(17)
O1	Te1	F6	178.26(6)	O4	Te4	F23	94.73(6)	F3	C3	C2	119.56(18)
O1	Te1	F10	94.53(6)	O4	Te4	F22	93.89(6)	C2	C3	C4	120.85(17)
F20	Te3	F17	88.84(5)	O4	Te4	F24	92.53(6)	F5	C5	N1	116.39(17)
F20	Te3	F18	174.75(5)	O4	Te4	F21	177.00(6)	F5	C5	C4	123.50(18)
F20	Te3	F19	90.82(6)	O2	Al1	O1	110.91(7)	N1	C5	C4	120.11(19)
F18	Te3	F17	89.03(6)	O3	Al1	O2	110.28(7)				
F19	Te3	F17	173.78(5)	O3	Al1	O4	109.15(7)				
F19	Te3	F18	90.78(6)	O3	Al1	O1	108.18(7)				
F16	Te3	F20	87.67(5)	O4	Al1	O2	108.60(7)				
F16	Te3	F17	87.00(5)	O4	Al1	O1	109.70(7)				
F16	Te3	F18	87.42(6)	Al1	O2	Te2	136.99(8)				
F16	Te3	F19	86.79(5)	Al1	O3	Te3	134.61(8)				
O3	Te3	F20	92.53(6)	Al1	O4	Te4	139.06(8)				
O3	Te3	F17	92.44(6)	Al1	O1	Te1	137.43(8)				
O3	Te3	F18	92.35(6)	C1	N1	C5	122.30(17)				
O3	Te3	F19	93.77(6)	F1	C1	N1	116.37(17)				
O3	Te3	F16	179.40(6)	F1	C1	C2	123.36(19)				
F12	Te2	F15	87.99(6)	N1	C1	C2	120.27(17)				
F12	Te2	F11	85.68(6)	F4	C4	C3	121.05(17)				
F11	Te2	F15	85.70(6)	F4	C4	C5	120.64(18)				
F13	Te2	F15	172.67(5)	C5	C4	C3	118.31(17)				

Table SI 2.4.1. Bond Lengths for $[(C_5Cl_5N)_2H][Al(OTeF_5)_4]\cdot C_6H_4F_2$ (**6a**$\cdot C_6H_4F_2$).

Atom	Atom	Length/Å	Atom	Atom	Length/Å
Te4	F19	1.8295(16)	Cl9	C9	1.703(2)
Te4	F20	1.8385(15)	Cl5	C5	1.713(3)
Te4	F18	1.8257(16)	Cl2	C2	1.704(3)
Te4	F17	1.8295(16)	Cl3	C3	1.707(2)
Te4	F16	1.8426(15)	Al1	O4	1.7528(18)
Te4	O4	1.8119(16)	Al1	O3	1.7407(19)
Te3	F13	1.8336(16)	Al1	O1	1.7264(19)
Te3	F14	1.8335(16)	Al1	O2	1.732(2)
Te3	F12	1.8278(16)	Cl4	C4	1.709(3)
Te3	F11	1.8343(17)	F22	C12	1.352(3)
Te3	O3	1.8110(18)	F21	C11	1.349(3)
Te3	F15	1.8248(17)	N2	C10	1.340(3)
Te1	F4	1.8289(17)	N2	C6	1.342(3)
Te1	F1	1.8355(16)	N1	C5	1.331(3)
Te1	F3	1.8215(19)	N1	C1	1.330(3)
Te1	O1	1.8024(18)	C7	C6	1.388(3)
Te1	F2	1.8218(19)	C7	C8	1.394(3)
Te1	F5	1.8189(19)	C10	C9	1.380(3)
Te2	F8	1.8207(17)	C5	C4	1.387(4)
Te2	F9	1.8368(17)	C8	C9	1.400(3)
Te2	F6	1.8270(17)	C2	C1	1.393(3)
Te2	F10	1.8255(18)	C2	C3	1.391(4)
Te2	F7	1.8185(18)	C4	C3	1.391(4)
Te2	O2	1.8128(19)	C11	C12	1.379(4)
Cl7	C7	1.700(2)	C11	C16	1.376(4)
Cl6	C6	1.692(2)	C12	C13	1.369(4)
Cl10	C10	1.689(2)	C14	C15	1.380(4)
Cl8	C8	1.695(2)	C14	C13	1.393(4)
Cl1	C1	1.715(3)	C16	C15	1.389(4)

Table SI 2.4.2. Bond Angles for $[(C_5Cl_5N)_2H][Al(OTeF_5)_4]\cdot C_6H_4F_2$ (**6a**$\cdot C_6H_4F_2$).

Atom	Atom	Atom	Angle/˚	Atom	Atom	Atom	Angle/˚	Atom	Atom	Atom	Angle/˚	Atom	Atom	Atom	Angle/˚
F19	Te4	F20	90.00(8)	O2	Te2	F9	92.17(9)	O1	Te1	F4	95.14(8)	F22	C12	C11	118.6(3)
F19	Te4	F17	172.45(7)	O2	Te2	F6	178.55(9)	O1	Te1	F1	178.58(9)	F22	C12	C13	120.4(3)
F19	Te4	F16	86.60(7)	O2	Te2	F10	93.51(9)	O1	Te1	F3	92.54(9)	C13	C12	C11	120.9(3)
F20	Te4	F16	85.91(7)	O2	Te2	F7	93.35(9)	O1	Te1	F2	91.24(9)	C10	C9	Cl9	120.13(19)
F18	Te4	F19	89.86(8)	O3	Al1	O4	108.29(9)	O1	Te1	F5	93.73(10)	C10	C9	C8	118.6(2)
F18	Te4	F20	173.16(7)	O1	Al1	O4	103.59(9)	F2	Te1	F4	173.59(8)	C8	C9	Cl9	121.30(19)
F18	Te4	F17	89.55(8)	O1	Al1	O3	113.28(10)	F2	Te1	F1	87.36(9)	C15	C14	C13	120.3(3)
F18	Te4	F16	87.26(8)	O1	Al1	O2	113.01(10)	F5	Te1	F4	88.85(10)	C11	C16	C15	119.0(3)
F17	Te4	F20	89.69(8)	O2	Al1	O4	111.37(9)	F5	Te1	F1	86.45(10)	C14	C15	C16	120.2(3)
F17	Te4	F16	85.85(7)	O2	Al1	O3	107.25(10)	F5	Te1	F3	173.68(9)	C12	C13	C14	118.9(3)
O4	Te4	F19	93.43(7)	Al1	O4	Te4	136.71(10)	F5	Te1	F2	90.15(11)				
O4	Te4	F20	93.79(7)	Al1	O3	Te3	136.12(11)	F8	Te2	F9	89.50(9)				
O4	Te4	F18	93.04(8)	Al1	O1	Te1	149.57(12)	F8	Te2	F6	87.53(8)				
O4	Te4	F17	94.11(8)	Al1	O2	Te2	140.72(12)	F8	Te2	F10	174.69(8)				
O4	Te4	F16	179.70(8)	C10	N2	C6	122.7(2)	F6	Te2	F9	86.61(8)				
F13	Te3	F11	87.13(8)	C1	N1	C5	118.7(2)	F10	Te2	F9	89.27(10)				
F14	Te3	F13	90.53(8)	C6	C7	Cl7	120.24(19)	F10	Te2	F6	87.25(9)				
F14	Te3	F11	86.76(8)	C6	C7	C8	118.4(2)	F7	Te2	F8	90.27(9)				
F12	Te3	F13	89.47(8)	C8	C7	Cl7	121.40(18)	F7	Te2	F9	174.48(9)				
F12	Te3	F14	173.77(8)	N2	C10	Cl10	116.46(18)	F7	Te2	F6	87.87(9)				
F12	Te3	F11	87.01(8)	N2	C10	C9	120.1(2)	F7	Te2	F10	90.46(10)				
O3	Te3	F13	93.15(8)	C9	C10	Cl10	123.42(19)	O2	Te2	F8	91.70(8)				
O3	Te3	F14	92.87(8)	N2	C6	Cl6	116.40(18)	C5	C4	Cl4	120.8(2)				
O3	Te3	F12	93.35(8)	N2	C6	C7	120.1(2)	C5	C4	C3	117.9(2)				
O3	Te3	F11	179.54(9)	C7	C6	Cl6	123.53(19)	C3	C4	Cl4	121.3(2)				
O3	Te3	F15	92.40(8)	N1	C5	Cl5	115.49(19)	N1	C1	Cl1	115.50(18)				
F15	Te3	F13	174.44(8)	N1	C5	C4	123.0(2)	N1	C1	C2	123.2(2)				
F15	Te3	F14	89.39(9)	C4	C5	Cl5	121.5(2)	C2	C1	Cl1	121.29(19)				
F15	Te3	F12	90.01(8)	C7	C8	Cl8	119.82(18)	C2	C3	Cl3	120.0(2)				
F15	Te3	F11	87.32(9)	C7	C8	C9	120.2(2)	C2	C3	C4	119.8(2)				
F4	Te1	F1	86.27(9)	C9	C8	Cl8	119.98(19)	C4	C3	Cl3	120.2(2)				
F3	Te1	F4	89.82(10)	C1	C2	Cl2	121.3(2)	F21	C11	C12	119.2(2)				
F3	Te1	F1	87.30(9)	C3	C2	Cl2	121.30(19)	F21	C11	C16	120.2(3)				
F3	Te1	F2	90.48(12)	C3	C2	C1	117.4(2)	C16	C11	C12	120.6(3)				

Table SI 2.5.1. Bond Lengths for [C_5F_4ClNH][$Al(OTeF_5)_4$] (**3a**).

Atom	Atom	Length/Å	Atom	Atom	Length/Å
Te1	F7	1.8294(15)	Al1	O1	1.7491(17)
Te1	F3	1.8397(14)	Al1	O1[1]	1.7490(17)
Te1	F4	1.8203(15)	Al1	O2	1.7225(18)
Te1	F5	1.8289(15)	Al1	O2[1]	1.7225(18)
Te1	O1	1.8086(15)	Cl04	C3	1.683(3)
Te1	F6	1.8350(15)	F1	C1	1.303(3)
Te2	F10	1.8191(15)	F2	C2	1.316(3)
Te2	F9	1.8216(16)	N1	C1	1.335(3)
Te2	F11	1.8218(16)	N1	C1[2]	1.335(3)
Te2	F8	1.8297(15)	C1	C2	1.365(3)
Te2	F12	1.8374(15)	C2	C3	1.390(3)
Te2	O2	1.7896(17)			

Table SI 2.5.2. Bond Angles for [C_5F_4ClNH][$Al(OTeF_5)_4$] (**3a**).

Atom	Atom	Atom	Angle/°	Atom	Atom	Atom	Angle/°	Atom	Atom	Atom	Angle/°
F7	Te1	F3	85.76(7)	F8	Te2	F12	86.41(8)	F9	Te2	F11	173.38(8)
F7	Te1	F6	89.01(8)	O2	Te2	F10	93.96(9)	F9	Te2	F8	86.43(8)
F4	Te1	F7	89.88(8)	O2	Te2	F9	91.90(8)	F9	Te2	F12	89.69(9)
F4	Te1	F3	87.52(7)	O2	Te2	F11	94.71(8)	F11	Te2	F8	86.96(8)
F4	Te1	F5	90.06(8)	O2	Te2	F8	178.20(9)	F11	Te2	F12	89.57(9)
F4	Te1	F6	173.00(7)	O2	Te2	F12	92.92(9)				
F5	Te1	F7	173.31(7)	O1[1]	Al1	O1	109.29(12)				
F5	Te1	F3	87.56(7)	O2	Al1	O1[1]	106.75(9)				
F5	Te1	F6	90.24(8)	O2[1]	Al1	O1[1]	108.85(8)				
O1	Te1	F7	92.50(7)	O2	Al1	O1	108.85(8)				
O1	Te1	F3	177.87(7)	O2[1]	Al1	O1	106.75(9)				
O1	Te1	F4	93.71(7)	O2[1]	Al1	O2	116.22(15)				
O1	Te1	F5	94.18(7)	Al1	O1	Te1	133.69(10)				
O1	Te1	F6	93.25(7)	Al1	O2	Te2	156.85(14)				
F6	Te1	F3	85.50(7)	C1	N1	C1[2]	121.6(3)				
F10	Te2	F9	90.40(8)	F1	C1	N1	116.8(2)				
F10	Te2	F11	89.55(8)	F1	C1	C2	122.7(2)				
F10	Te2	F8	86.72(8)	N1	C1	C2	120.5(2)				
F10	Te2	F12	173.12(8)	F2	C2	C1	119.8(2)				

III Hirshfeld Plots

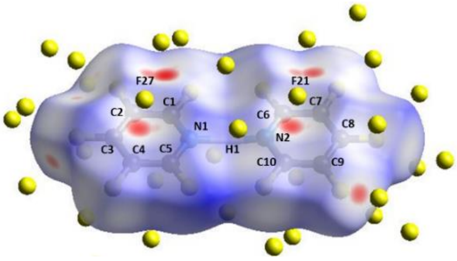

Figure SI 3.1. Hirshfeld surface of the $[(C_5F_5N){\cdot\cdot}H(NC_5F_5)]$ moiety of $[(C_5F_5N)_2H][Al(OTeF_5)_4]$ in the solid state. Complete anion fragments are omitted for clarity. Red contours indicate the areas where close-contact interactions are observed.

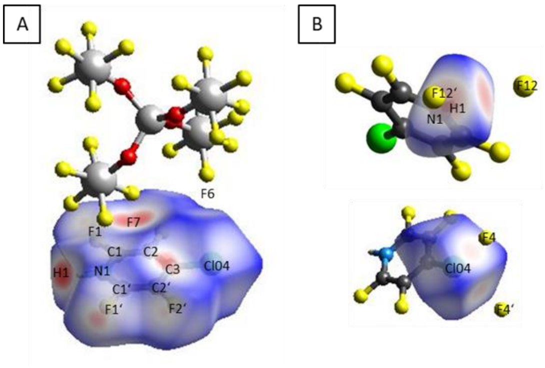

Figure SI 3.2. Hirshfeld surface of the structure of $[C_5F_4ClNH][Al(OTeF_5)_4]$ in the solid state. A – interaction between cation and anion; B – interaction between H1 and F12/F12'(top); between Cl4 and F4/F4' (bottom). The red contours indicate the areas where close-contact interactions are observed.

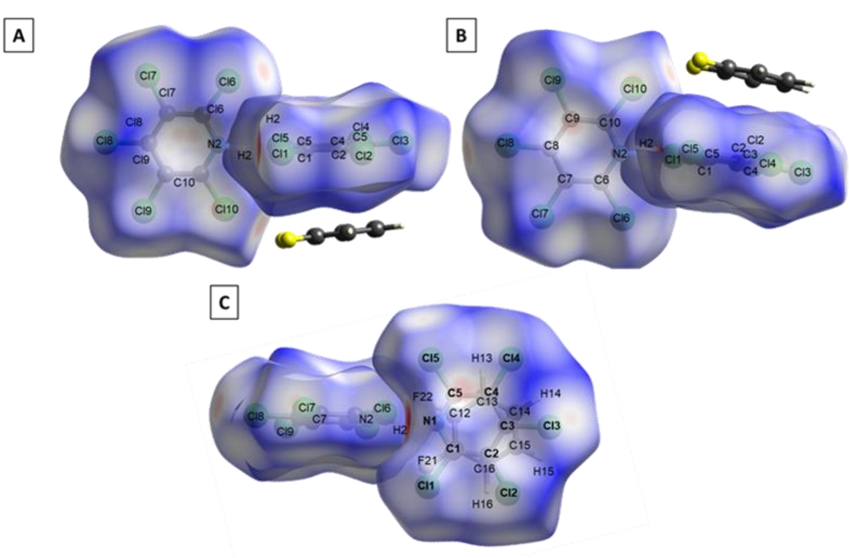

Figure SI 3.3. Hirshfeld surface of the $[(C_5Cl_5N)_2H]^+$ cation in the solid state structure of $[(C_5F_5N)_2H][Al(OTeF_5)_4]$. Anion is omitted for clarity. A and B – front view with co-crystalized *ortho*-difluorobenzene; C – top view with co-crystalized *ortho*-difluorobenzene hidden behind the C_5Cl_5NH moiety. Red contours indicate the areas where close-contact interactions are observed.

IV IR spectra

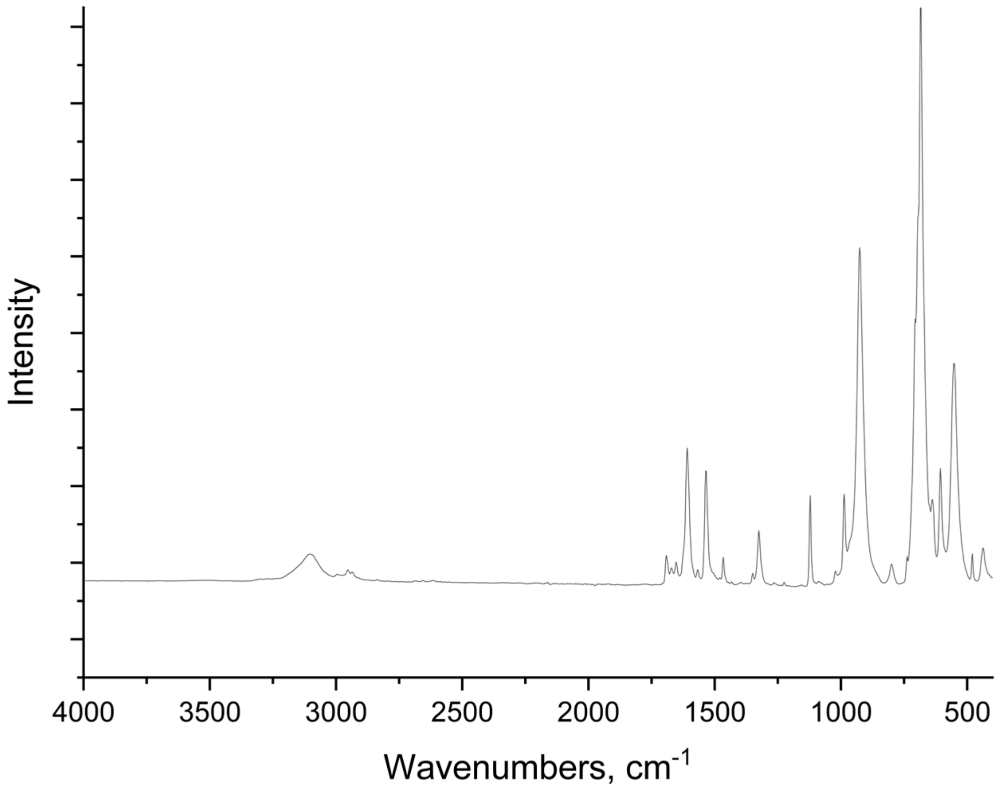

Figure SI 4.1. Experimental IR spectrum of $[C_5F_5NH][Al(OTeF_5)_4]$ (**2a**).

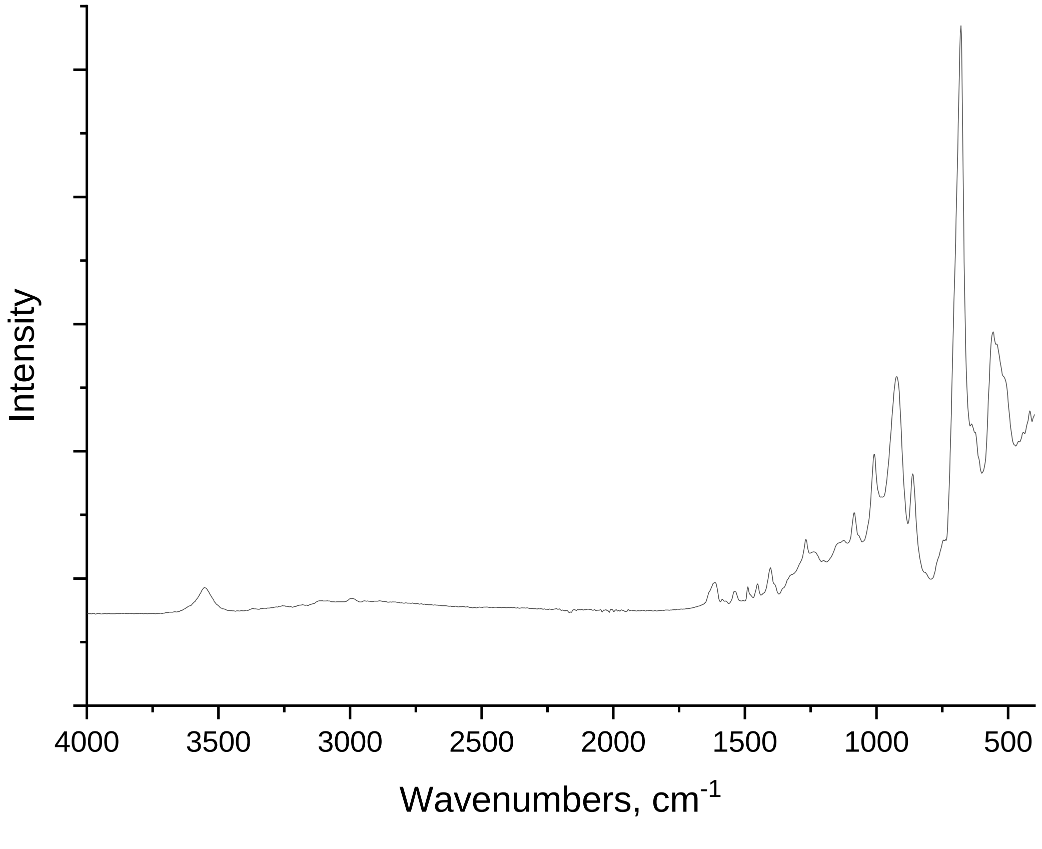

Figure SI 4.2. Experimental IR spectrum of $[(C_5Cl_5N)_2H][Al(OTeF_5)_4]$ (**6a**).

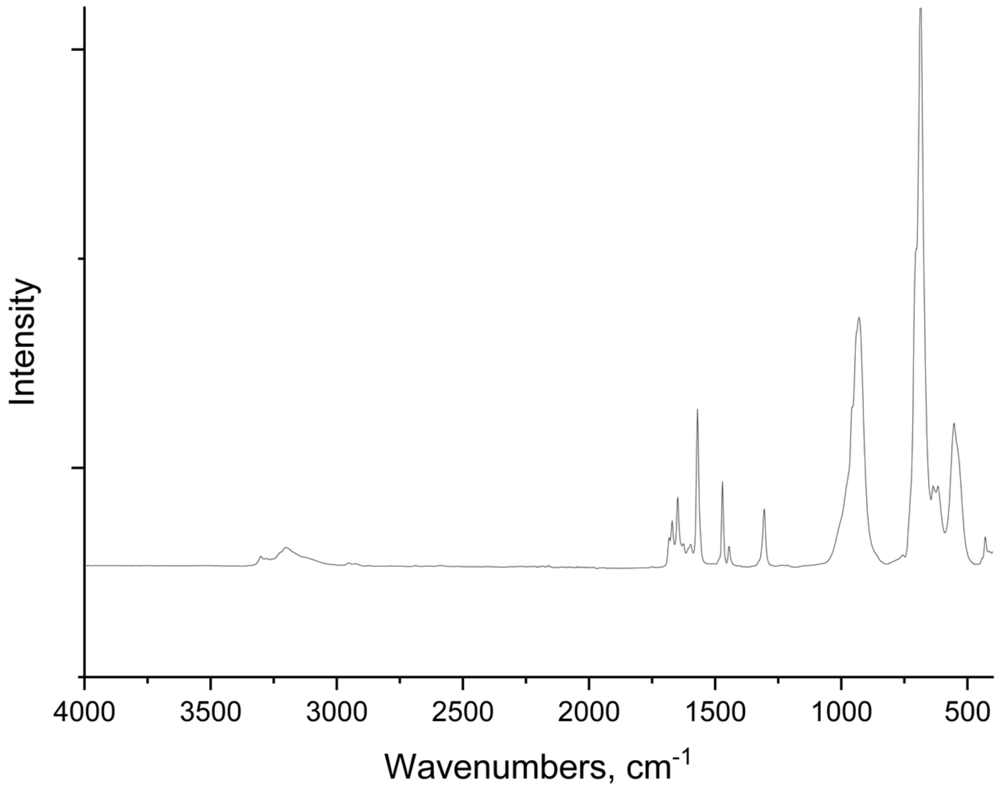

Figure SI 4.3. Experimental IR spectrum of $[C_5F_4ClNH][Al(OTeF_5)_4]$ (**3a**).

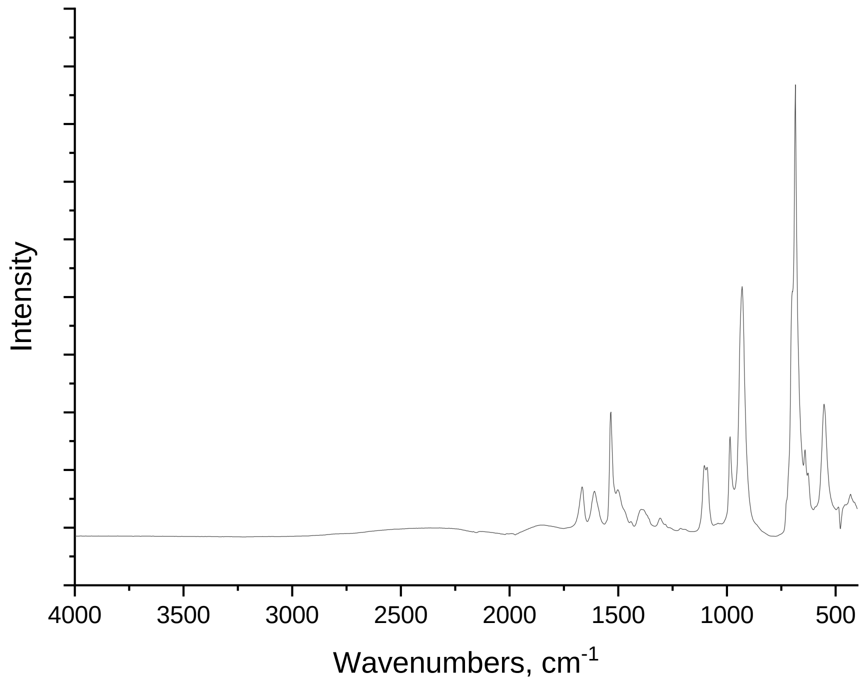

Figure SI 4.4. Experimental IR spectrum of $[(C_5F_5N)_2H][Al(OTeF_5)_4]$ (**5a**).

V. Quantum-Chemical Calculations

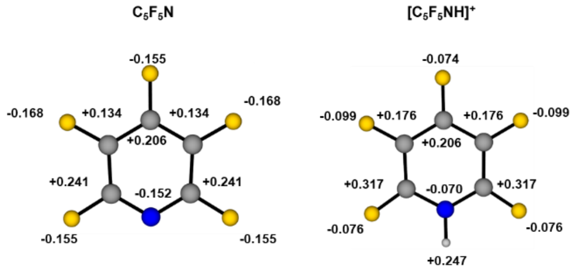

Figure SI 5.1. Mulliken partial charges of neutral C_5F_5N (left) and the $[C_5F_5NH]^+$ cation (right), calculated on B3LYP-D3/def2-TZVPP level of theory.

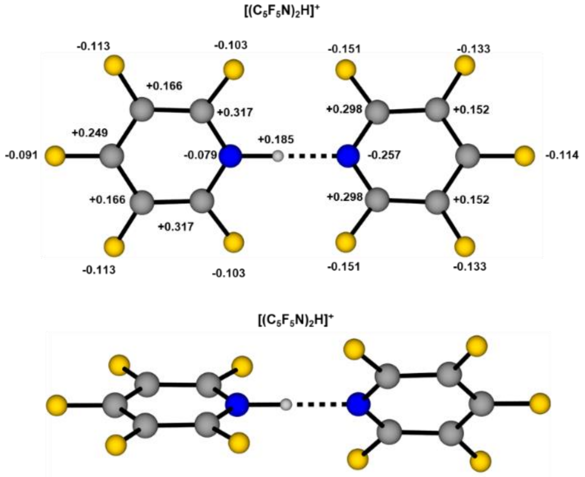

Figure SI 5.2. Mulliken partial charges of the minimum structure of $[(C_5F_5N)_2H]^+$ (top, top view and bottom, side view) with α(C-N-N-C) dihedral angle of 125°), calculated on B3LYP-D3/def2-TZVPP level of theory.

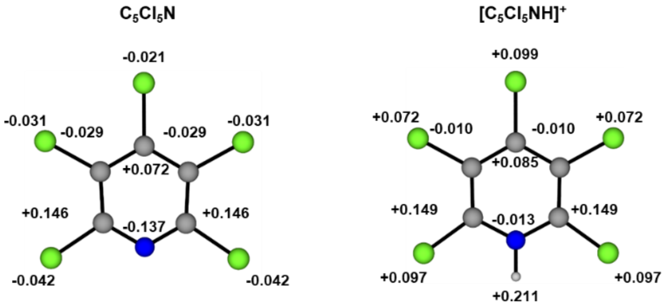

Figure SI 5.3. Mulliken partial charges of neutral C_5Cl_5N (left) and the $[C_5Cl_5NH]^+$ cation (right), calculated on B3LYP-D3/def2-TZVPP level of theory.

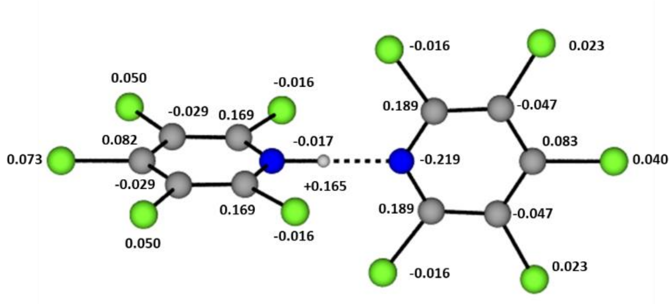

Figure SI 5.4. Mulliken partial charges of the minimum structure of $[(C_5Cl_5N)_2H]^+$ (top, top view and bottom, side view) with α(C-N-N-C) dihedral angle of 90°), calculated on B3LYP-D3/def2-TZVPP level of theory.

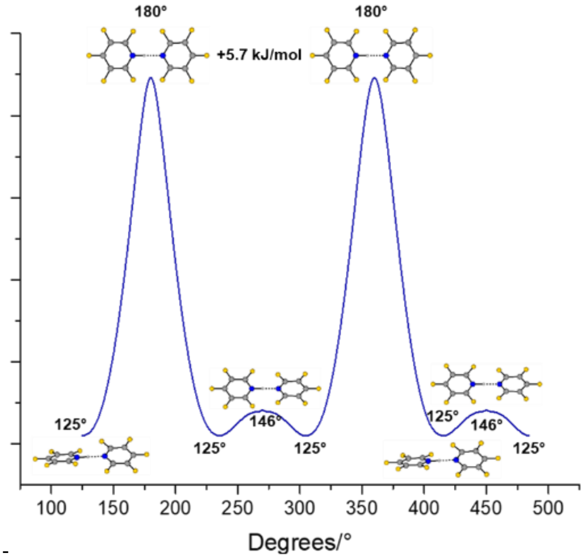

-

Figure SI 5.5. Relaxed potential energy scan (RPES) of the rotation of C_5F_5N around the torsional dihedral angle coordinate α (∠C-N-N-C) in the $[(C_5F_5N)_2H]^+$ dimer starting from the minimum structure with an α (∠C-N-N-C) dihedral angle of 125°, calculated on B3LYP-D3/def2-TZVPP level of theory. No restrictions were imposed while calculating RPES.

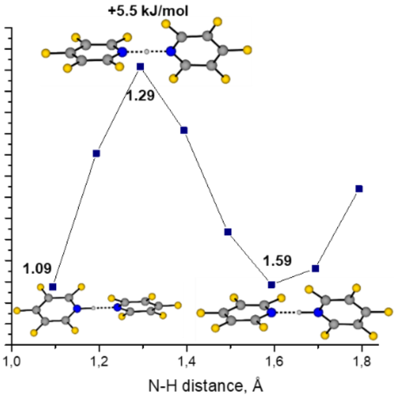

Figure SI 5.6. Potential energy scan of the N-H distance in the $[(C_5F_5N)_2H]^+$ dimer starting from the minimum structure, calculated on B3LYP-D3/def2-TZVPP level of theory. N-N distance was frozen in redundant coordinates while calculating PES.

Table SI 5.1. Coordinates for the gas phase optimized structures of halogenated pyridinium dimers (B3LYP-D3/def2-TZVPP level of theory, T= 20°C).

**Co-planar $[(C_5F_5N)_2H]^+$**				**Minimum $[(C_5F_5N)_2H]^+$**			
1 1				1 1			
C	2.07726400	1.16954100	0.00020300	C	2.01884700	1.03556400	-0.54136800
C	3.45817200	1.20956900	0.00052600	C	3.39961700	1.07435000	-0.56105400
C	4.15644600	-0.00043300	0.00024000	C	4.09576500	-0.00036700	0.00000500
C	3.45746100	-1.21000900	-0.00035800	C	3.39894900	-1.07452900	0.56131600
C	2.07657500	-1.16911800	-0.00065100	C	2.01821100	-1.03463100	0.54210200
H	0.33810000	0.00045700	-0.00040900	H	0.27022200	0.00105900	0.00048300
N	1.41489300	0.00040300	-0.00040400	N	1.36377200	0.00073200	0.00047800
F	1.35904100	-2.25452700	-0.00123400	F	1.28622100	-1.99037700	1.04491400
F	4.09312900	-2.35866700	-0.00064200	F	4.03756100	-2.09121000	1.09272000
F	5.45893700	-0.00081100	0.00053500	F	5.39903800	-0.00088600	-0.00021300
F	4.09454600	2.35783100	0.00110600	F	4.03885500	2.09052800	-1.09267400
F	1.36043200	2.25540700	0.00048100	F	1.28746100	1.99189300	-1.04393800
C	-2.07800900	-1.13632400	0.00016300	C	-2.01805000	-1.00817600	-0.53176600
C	-2.07723000	1.13577900	-0.00054000	C	-2.01994700	1.00956600	0.53187400
C	-3.46117100	-1.20204800	0.00046600	C	-3.40060200	-1.06759600	-0.56197700
C	-3.46032400	1.20253000	-0.00027000	C	-3.40263700	1.06668000	0.56131400
C	-4.16496300	0.00048300	0.00024000	C	-4.10273300	-0.00102100	-0.00051600
N	-1.39853000	-0.00051000	-0.00035800	N	-1.34991100	0.00123200	0.00023600
F	-1.37110400	-2.25508200	0.00042500	F	-1.29315400	-1.98765100	-1.04847300
F	-4.09550000	-2.35967900	0.00097500	F	-4.03708200	-2.09003800	-1.10003000
F	-5.47596800	0.00094600	0.00051800	F	-5.41322300	-0.00208500	-0.00087400
F	-4.09383200	2.36061000	-0.00048600	F	-4.04107600	2.08808000	1.09902200
F	-1.36945600	2.25402500	-0.00105300	F	-1.29701900	1.99020600	1.04898500

**Co-planar $[(C_5Cl_5N)_2H]^+$**				**Minimum $[(C_5Cl_5N)_2H]^+$**			
1 1				1 1			
C	2.54135100	-1.09980100	0.00000000	C	-2.00580500	0.86823300	-0.79969000
C	3.91888200	-1.29258800	0.00000000	C	-3.39753200	0.89879500	-0.82816400
C	4.75667600	-0.15961400	0.00000000	C	-4.09930600	-0.00005400	0.00001200
C	4.19853500	1.13548100	0.00000000	C	-3.39745300	-0.89878600	0.82824800
C	2.81288300	1.26604100	0.00000000	C	-2.00572900	-0.86799100	0.79989500
N	2.03832100	0.15544400	0.00000000	N	-1.36865100	0.00017300	0.00012800
Cl	2.02319100	2.76156200	0.00000000	Cl	-4.21361900	-2.00079400	1.84378700
Cl	5.18580000	2.52572100	-0.00000100	Cl	-5.79839100	-0.00019500	-0.00006100
Cl	6.44050900	-0.35463700	0.00000000	Cl	-4.21379300	2.00066900	-1.84377100
Cl	4.55601300	-2.87363300	0.00000100	H	-0.28847600	0.00026600	0.00017900
Cl	1.40780500	-2.35264100	0.00000100	C	2.00583800	0.78254700	0.84343600
C	-2.78669200	-1.20779600	0.00000000	C	2.00557900	-0.78242400	-0.84340000
C	-2.56099300	1.05824900	0.00000000	C	3.40025900	0.82740600	0.89170400
C	-4.18529700	-1.13422800	0.00000000	C	3.39998600	-0.82753100	-0.89186600
C	-3.94045800	1.27790600	0.00000000	C	4.10564300	-0.00012500	-0.00013100
C	-4.76674500	0.14329700	0.00000000	N	1.34887000	0.00012000	0.00006400
N	-2.01330400	-0.13843800	0.00000000	Cl	4.21469800	1.85124900	1.99503700
Cl	-1.45217000	2.39602900	0.00000000	Cl	5.81205300	-0.00027700	-0.00025100
Cl	-4.59814300	2.86217400	0.00000000	Cl	4.21408700	-1.85151900	-1.99531400
Cl	-6.46866300	0.31807700	-0.00000100	Cl	1.02593700	-1.74924600	-1.88574300
Cl	-5.15723100	-2.54744800	0.00000000	Cl	1.02651600	1.74954400	1.88591700
Cl	-2.00339600	-2.75338400	0.00000000	Cl	-1.02313100	-1.88951100	1.74093200
H	1.02284900	0.26834800	0.00000000	Cl	-1.02329600	1.88991900	-1.74064000

VI References

[1] S. Berger, S. Braun, H.-O. Kalinowski, *NMR-Spektroskopie von Nichtmetallen*. *^{19}F-NMR-Spektroskopie*, Georg Thieme, Stuttgart, New York, **1994**.
